# Guard cell signal transduction mechanisms controlling stomatal movements by light, abscisic acid, CO_2_, VPD and heat

**DOI:** 10.1093/plphys/kiag653

**Published:** 2026-09-04

**Authors:** Julian I Schroeder, Toshinori Kinoshita, Nobuyuki Uozumi, Sarah M Assmann

**Affiliations:** Cell and Developmental Biology Department, School of Biological Sciences, University of California San Diego, 9500 Gilman Drive, La Jolla, CA 92093-0116, United States; Institute of Transformative Bio-Molecules (WPI-ITbM), Nagoya University, Chikusa, Nagoya 464-8602, Japan; Department of Biomolecular Engineering, Graduate School of Engineering, Tohoku University, Aobayama 6-6-07, Sendai 980-8579, Japan; Department of Biology, Penn State University, University Park, PA 16802-5301, United States

## Abstract

Guard cell pairs form dynamic stomatal gas exchange pores in aerial tissues of plants. Guard cells regulate CO_2_ influx and water loss through stomata by integrating multiple environmental and internal signals. Guard cells serve as highly specialized sensory and signal processing cells, thereby orchestrating stomatal movements in response to changing conditions. This review describes recent advances and early discoveries in elucidation of guard cell signaling networks. Present day understanding of the molecular and cellular mechanisms mediating stomatal opening by blue light, red light, heat stress and photosynthesis-mediated low CO_2_ in intercellular leaf spaces are reviewed. Furthermore, up-to-date knowledge of the molecular mechanisms and pathways that trigger stomatal closing by abscisic acid (ABA), elevated CO_2_ and low humidity/high vapor pressure difference is synthesized. These pathways promote stomatal closure and water conservation. Critical functions of other tissues are described, including mesophyll cells as producers of messengers for red light-induced stomatal opening and vascular tissues in drought-induced ABA synthesis and transport. Guard cell ion channels and pumps drive the ion fluxes required for the cellular osmotic motor that opens and closes stomatal pores. Roles of and recent advances toward understanding the regulatory mechanisms of these membrane transporters as downstream targets of guard cell signaling cascades are described. Synthesis of these signaling cascades into signaling networks is reviewed and open questions for future research are highlighted throughout. Understanding these integrated mechanisms is critical for future improvement of plant water use efficiency and environmental stress resilience of crops.

## Introduction

Stomata respond rapidly to changing environmental conditions by opening and closing stomatal pores. Blue light triggers stomatal opening by activating photoreceptors, the phototropins (Huala et al. 1997; Christie et al. 1998; Kinoshita et al. 2001), that activate proton pumps (Assmann et al. 1985; Shimazaki et al. 1986) and regulate ion channels in guard cell membranes. This results in potassium and anion ion uptake into guard cells and an increase in organic solutes, resulting in osmotic water influx into guard cells that cause turgor and volume increases thereby opening stomatal pores due to mechanical constraints from cell walls. Red light promotes stomatal opening through a combination of guard cell and whole leaf responses linking photosynthesis and mesophyll-derived signals to stomatal opening (Roelfsema et al. 2002; Zait et al. 2025). A reduction in the CO_2_ concentration in leaves caused by photosynthesis triggers stomatal opening (Raschke 1975a, 1975b; Assmann 1999; Roelfsema et al. 2002), as does an increase in atmospheric humidity (Hõrak et al. 2017; Merilo et al. 2018; Buckley 2019). Heat stress triggers rapid opening of stomatal pores, which facilitates transpirational cooling of leaves (Darwin 1898; Kostaki et al. 2020; Pankasem et al. 2024).

In contrast to the above stomatal opening stimuli, drought induces the synthesis of the plant hormone abscisic acid (ABA), which triggers ion efflux and water loss from guard cells, causing stomatal closure and thus conserving water (MacRobbie 1981). Other stimuli also play key roles in triggering stomatal closing. A transition from light to darkness causes a rapid stomatal closing response that is likely caused by a combination of mechanisms: Removal of blue and red light stimuli reverses their actions, thus promoting stomatal closure. Furthermore, darkness rapidly causes elevated CO_2_ concentrations in leaf intercellular air spaces (Hanstein et al. 2001) due to respiration and lack of photosynthesis. Together, these responses close stomata, thereby curtailing transpiration when the photosynthetic demand for CO_2_ is absent. Moreover, the rising atmospheric CO_2_ concentration is causing additional reduction in stomatal apertures throughout the entire day-night cycle with CO_2_ elevation during the present century predicted to reduce stomatal pore apertures by up to 40% (Medlyn et al. 2001; Franks et al. 2017).

Reductions in atmospheric humidity increase the vapor pressure difference (VPD_leaf_) from intercellular air spaces in leaves to the air in the vicinity of leaves. This results in stomatal closure, reducing water loss via transpiration. Interestingly, heat stress can have opposing effects depending on the conditions: (ⅰ) Heat causes stomatal opening, which enables transpirational cooling. (ⅱ) However, heat can cause an increase in VPD_leaf_—for thermodynamic reasons—resulting in stomatal closing. Thus, heat can cause either stomatal opening or stomatal closing, dependent on relative humidity and other environmental parameters.

Many but not all of the above signal transduction mechanisms take place in guard cells, with other leaf cells, mesophyll, epidermal pavement cells and the vasculature contributing to these responses, as will be reviewed. Together, the above stimuli trigger signaling pathways that are integrated in guard cells to optimize CO_2_ uptake for photosynthesis, while regulating plant water loss. The present understanding of how these sensor/receptor signal transduction pathways regulate ion channels, pumps, co-transporters and other mechanisms will be reviewed here. Research on plant pathogens that modulate stomatal apertures has recently been reviewed elsewhere (Wu and Liu 2022; Chen and Torii 2023; Melotto et al. 2024).

Guard cells regulate stomatal apertures through the coordinated activities of ion channels, pumps, and transporters in the plasma membrane and endosomal membranes. During stomatal opening, plasma membrane H^+^-extruding ATPases generate an electrochemical gradient that drives potassium (K^+^) uptake through inward-rectifying K^+^ channels (Schroeder et al. 1987).

The pH gradient and negative membrane potential together drive anion uptake and sucrose uptake mediated by H^+^-coupled co-transporters. In contrast, stomatal closing involves a reduction in H^+^ ATPase activity concomitant with activation of S-type and R-type anion channels and Ca^2+^-permeable channels in the plasma membrane, leading to more positive (less negative) membrane potentials (depolarization), resulting in efflux of K^+^ through outward-rectifying K^+^ channels, and osmotic water loss from guard cells. Parallel vacuolar ion transport systems further play central roles in regulating guard cell turgor (MacRobbie 1981), as ion transport mechanisms in the vacuolar membrane respond to changes in cytosolic messengers and solute concentrations engendered by transport activities at the plasma membrane. Vacuoles can take up 90% of the guard cell volume. During stomatal opening, V-type H^+^-ATPases energize the vacuolar membrane, supporting vacuolar ion accumulation through transporters and channels such as ALMTs and K^+^/H^+^ exchangers, as reviewed elsewhere (Martinoia et al. 2012). During stomatal closing, vacuolar cation release is regulated and largely mediated by VK channels and SV channels (Ward and Schroeder 1994; Martinoia et al. 2012). The *TPC1* gene was found to encode SV channels in plants with evidence for a role of SV channels in Ca^2+^-induced stomatal closing (Peiter et al. 2005). Vacuolar ion transport and other important topics, including the roles of the guard cell wall, cytoskeleton, and endomembrane system in stomatal movements as well as characterization of the dynamic guard cell transcriptome, metabolome, and proteome, are not covered here due to space constraints, but are the subject of other excellent reviews (Medeiros et al. 2015; Misra et al. 2015; Daloso et al. 2017; Rui et al. 2018; Li et al. 2022a, 2022b; Koiwa et al. 2026).

In the present review, we primarily focus on guard cell signaling pathways and regulatory mechanisms at the plasma membrane. In many studies the rapid stimulus-triggered regulation of plasma membrane channels and pumps has been used to dissect early signal transduction mechanisms, including in many time-resolved single cell studies. Identification and characterization of these signaling pathways is advancing our understanding of the mechanisms underlying plant responses to environmental changes.

## Blue light signaling in guard cells: from photoreceptors to plasma membrane H^+^-ATPase activation, anion channel inhibition and starch degradation

The regulation of stomatal opening by light was first recognized by Francis Darwin, the son of Charles Darwin, who observed that stomata in leaves positioned near a window were open, whereas those in leaves farther from the window remained closed (Darwin 1898). This seminal observation laid the foundation for later studies on stomatal photobiology and ultimately led to the identification of blue light as a key signal controlling guard cell function. Blue light is a potent environmental signal promoting stomatal opening. In guard cells, blue light induces rapid membrane hyperpolarization, K^+^ influx, water uptake, and ultimately stomatal opening (Shimazaki et al. 2007). This response is mediated by a well-characterized signaling cascade that connects the blue light photoreceptor, phototropin, with a direct downstream component BLUS1 (Blue Light Signaling 1) kinase to the activation of the plasma membrane (PM) H^+^-ATPase (Kinoshita and Shimazaki 1999; Kinoshita et al. 2001; Takemiya et al. 2013) (Fig. 1). Over the past decade, significant progress has been made in elucidating the molecular components of this pathway, including a Raf-like protein kinase BHP (Blue light-dependent H^+^-ATPase Phosphorylation) mediating activation of the PM H^+^-ATPase (Hayashi et al. 2017), and distinct Raf-like protein kinases CBC (Convergence of Blue light and CO_2_) 1/2 responsible for the inhibition of S-type anion channels (Hiyama et al. 2017) (Fig. 1). Furthermore, early studies provided important insights into the metabolic basis of stomatal opening. In particular, Ogawa et al. (1978) demonstrated that red and blue light act synergistically to promote malate formation in guard cells, highlighting the light-dependent regulation of organic acid metabolism. Building on these findings, subsequent work by Talbott and Zeiger (1993) established that starch degradation and the accumulation of malate and sugars provide osmotic solutes that contribute to stomatal opening. Extending this classical framework, evidence indicates that phototropin signaling induces starch degradation and stored triacylglycerol breakdown, which are involved in the promotion of stomatal opening (Horrer et al. 2016; McLachlan et al. 2016). Notably, a recent study suggests that starch degradation is not universally required for blue light-induced stomatal opening across plant species (Bahadar et al. 2025).

**Figure 1:**
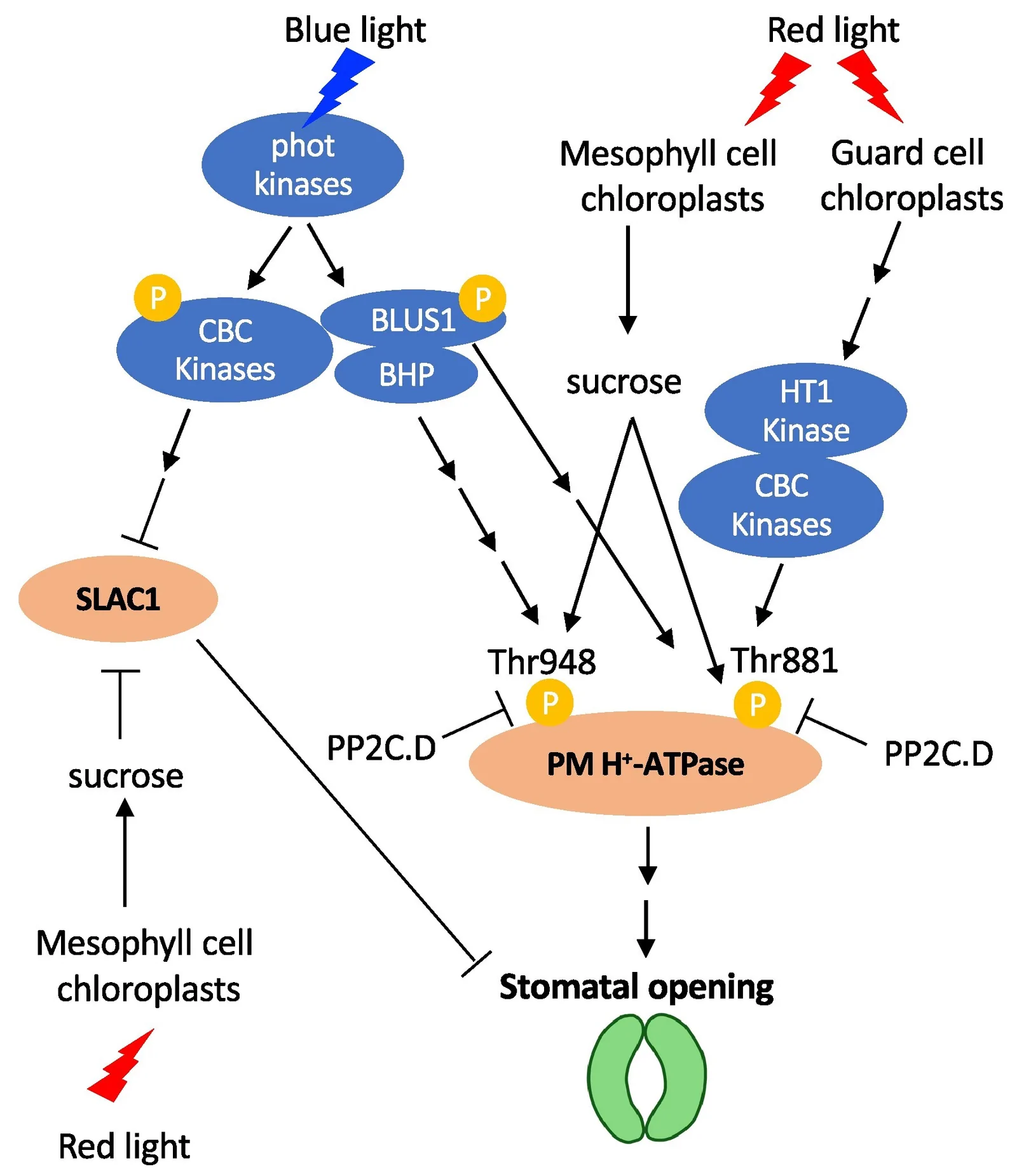
Light-induced stomatal opening signaling. Upon blue light perception, phototropins (phot kinases) phosphorylate the BLUS1 kinase and CBC kinases. BLUS1 indirectly induces phosphorylation and activation of the plasma membrane (PM) H^+^-ATPase at the C-terminal sites, Thr881 and Thr948, through signaling pathways that remain incompletely understood, thereby generating the driving force for stomatal opening. The protein kinase BHP interacts with BLUS1 and functions as a signaling component involved in the regulation of Thr948 phosphorylation. In parallel, CBC kinases inhibit the S-type anion channel SLAC1, thereby reducing anion efflux and promoting stomatal opening. Red light stimulates stomatal opening through both guard cell- and mesophyll-dependent pathways. In guard cells, photosynthetic activity promotes phosphorylation of the PM H^+^-ATPase at Thr881. This phosphorylation event is mediated by a heterodimer formed between HT1 and CBC kinases, with CBC1 directly phosphorylating Thr881. In mesophyll cells, photosynthesis produces sucrose, which acts as a mesophyll messenger that promotes PM H^+^-ATPase activation and inactivation of the S-type anion channel SLAC1 in guard cells. The type 2C protein phosphatases, clade D (PP2C.D), negatively regulate the PM H^+^-ATPase activity through dephosphorylation of Thr881 and Thr948. Black arrows indicate positive regulation, whereas T-shaped lines indicate inhibition.

With regard to the activation of the PM H^+^-ATPase, a well-established phosphorylation of Thr948 in the C-terminal autoinhibitory domain generates a binding motif for 14-3-3 proteins, followed by the binding of 14-3-3 proteins that relieves autoinhibition and stabilizes the H^+^-ATPase in its active state (Svennelid et al. 1999). (Amino acid numbering of Thr948 and other residues are according to the major Arabidopsis PM H^+^-ATPase AHA1). In addition, recent studies have revealed that phosphorylation of Thr881 in the same C-terminal region, which depends on both phototropin signaling and guard cell photosynthesis, is also important (Fuji et al. 2024; Hayashi et al. 2024). Several candidate kinases and phosphatases involved in these phosphorylation events have been proposed, and they are described in the following section.

### Blue light photoreceptors and initiation of signaling

In higher plants, phototropins function as the primary blue light receptors in guard cells. A major conceptual advance came from the work of Sarah Assmann and colleagues, who demonstrated that blue light specifically induces rapid membrane hyperpolarization and electrogenic ion pumping in guard cells, independently of photosynthetic red light responses (Assmann et al. 1985). This physiological framework was further strengthened by pioneering studies from Ken-ichiro Shimazaki and Eduardo Zeiger showing blue light-dependent proton extrusion (Shimazaki et al. 1986), ultimately leading to the molecular identification of phot1 and phot2 in *Arabidopsis thaliana* as the receptors mediating this response (Kinoshita et al. 2001). These phototropins are serine/threonine kinases containing 2 N-terminal LOV domains and a C-terminal kinase domain phototropins (Huala et al. 1997; Christie et al. 1998; Kinoshita et al. 2001). Upon blue light absorption, phototropins undergo auto-phosphorylation, thereby initiating downstream signaling events that culminate in stomatal opening. Genetic and physiological analyses further demonstrated that phot1 and phot2 function redundantly (Kinoshita et al. 2001). Thus, phototropin activation is considered the earliest defined molecular event in blue light-specific stomatal opening (Inoue et al. 2008). However, phototropins themselves do not directly phosphorylate the PM H^+^-ATPase, suggesting the existence of intermediate signaling components that transmit the signal from photoreceptors to the PM H^+^-ATPase.

### BLUS1 as a specific signaling component in guard cells

A major breakthrough in the field was the identification of BLUS1, a protein kinase specifically expressed in guard cells, as a key enzyme acting downstream of phototropins. BLUS1 is phosphorylated by phototropins in a blue light-dependent manner and is essential for PM H^+^-ATPase activation and stomatal opening (Takemiya et al. 2013). Loss-of-function *blus1* mutants show severely impaired blue light-induced stomatal opening despite normal phototropin activation based on Far-Western blot detection of 14-3-3 protein binding to the blue light-induced phosphorylated phototropin, indicating that BLUS1 functions as an intermediate signaling component in the blue light pathway in guard cells. Subsequent studies revealed that BLUS1 possesses kinase activity and is regulated by autoinhibitory domains at its C-terminus (Hosotani et al. 2021). BLUS1 does not appear to directly phosphorylate the H^+^-ATPase, but instead transmits the signal to additional downstream components. Although the precise mechanisms remain to be fully elucidated, candidates include a Raf-like protein kinase BHP and type 1 protein phosphatase, both of which have been implicated in the regulation of H^+^-ATPase activation and stomatal opening (Takemiya et al. 2006; Hayashi et al. 2017). Intriguingly, BHP itself does not directly phosphorylate the H^+^-ATPase but forms a complex with BLUS1 (Hayashi et al. 2017), suggesting that the signal is relayed through a multiprotein complex, the full composition and mode of action of which remain to be elucidated.

### CBC kinases and regulation of S-type anion channels

In parallel with H^+^-ATPase activation, blue light signaling also suppresses anion efflux from guard cells. Anion channels are central controllers of stomatal closure, integrating multiple signaling inputs to control membrane depolarization and solute efflux. S-type anion channels (Schroeder and Hagiwara 1989), encoded by SLAC1 (Slow Anion Channel 1) and SLAH3 (Negi et al. 2008; Vahisalu et al. 2008) mediate sustained anion release and stomatal closing (Schroeder et al. 1993; Schwartz et al. 1995). S-type anion channels are activated by ABA and CO_2_ signaling pathways. CBC1 and CBC2 were identified as Raf-like protein kinases that function downstream of phototropins (Hiyama et al. 2017). Based on electrophysiological analyses using patch-clamp recordings from guard cell protoplasts, which revealed that blue light suppresses S-type anion channel activity in wild-type plants but not in the *cbc1 cbc2* double mutant, CBC kinases are required for blue light-induced inhibition of S-type anion channels (Hiyama et al. 2017). This mechanism and other targets of the CBC kinases prevent membrane depolarization and support sustained hyperpolarization mediated by the PM H^+^-ATPase (Hiyama et al. 2017) (Fig. 1). CBC kinases thus represent a key node at which blue light and CO_2_ signaling converge. However, the roles of CBC1 and CBC2 in guard cells remain unresolved, as apparently contrasting functions have been reported. In addition to their roles in promoting blue light signaling, CBC1 and CBC2 have been proposed to act as negative regulators of blue light-induced activation of the H⁺-ATPase and stomatal opening ( Hayashi et al. 2020).

### Blue light-induced activation of the PM H^±^-ATPase in guard cells

The PM H^+^-ATPase is the ultimate effector of blue light-induced stomatal opening. Activation of this pump leads to proton extrusion, membrane hyperpolarization, and subsequent K^+^ influx through inward-rectifying K^+^ channels in guard cells (Shimazaki et al. 2007; Inoue and Kinoshita 2017). This sequence of events is supported by classical electrophysiological studies demonstrating blue light-induced electrogenic ion pumping (Assmann et al. 1985) and the voltage dependence and transport properties of K^+^ channels in guard cells (Schroeder et al. 1987). Activation of the PM H^+^-ATPase is tightly regulated by phosphorylation of a conserved penultimate threonine residue (Thr948) and a conserved Thr881 in the C-terminal autoinhibitory domain (Fig. 1). The kinase responsible for phosphorylation of Thr948 had long remained unknown, although in vitro kinase activity targeting the Thr948 of the PM H^+^-ATPase was detected in plasma membrane fractions from spinach leaves, microsomes of guard cell protoplasts from fava bean, and plasma membranes of etiolated Arabidopsis seedlings (Svennelid et al. 1999; Hayashi et al. 2010). This “missing kinase” represents one of the most important unresolved questions in blue light signaling. A MAP4K4/TOT3 kinase has been shown to directly phosphorylate Thr948 in vitro linked to reported high temperature-induced stomatal opening (Xu et al. 2025), but a TOT3 involvement in blue-light responses remains unknown. Takeshi Umezawa and colleagues demonstrated that HT1 (High Temperature 1) forms a heterodimer with the Raf-like kinase CBC1, in which HT1 activates the kinase activity of CBC1. Activated CBC1 then directly phosphorylates Thr881 of the plasma membrane H^+^-ATPase *in planta* (Takase et al. 2026).

In guard cells, the phosphatase responsible for dephosphorylation of Thr948 of the PM H^+^-ATPase was initially suggested, based on biochemical evidence, to exhibit type 2C protein phosphatase (PP2C)-like activity (Hayashi et al. 2010). Later studies identified PP2C clade D (PP2C.D) phosphatases as key regulators of this dephosphorylation process (Wong et al. 2021; Akiyama et al. 2022). More recently, PP2C.Ds have also been shown to catalyze dephosphorylation of phosphorylated Thr881 (Hayashi et al. 2024). How PP2C.D activity is regulated remains a major open question. In hypocotyl cells, SAUR proteins are known as negative regulators of PP2C.D and play a role in auxin-induced activation of the PM H^+^-ATPase (Spartz et al. 2014), and it has been suggested that SAURs also function in guard cells (Wong et al. 2021).

### Stomatal UV responses

In addition to blue and red light, UV-B has emerged as an important regulator of stomatal movements. The identification of UV RESISTANCE LOCUS8 (UVR8) as the UV-B photoreceptor established a dedicated UV-B signaling pathway in plants (Rizzini et al. 2011). Subsequent biochemical and structural studies revealed that UVR8 directly perceives UV-B through intrinsic tryptophan residues without the requirement for an external chromophore (Christie et al. 2012; Wu et al. 2012). Early physiological studies reported that UV-B can either promote stomatal opening or induce stomatal closure depending on the irradiation conditions and physiological status of the plant (Jansen and Van Den Noort 2000). More recently, Liu et al. demonstrated that UVR8 directly interacts with the lipoxygenase LOX1, activating a LOX1-dependent oxylipin and nitric oxide signaling pathway that promotes stomatal closure (Liu et al. 2025). Furthermore, UV-B signaling interacts with other light-quality pathways to fine-tune stomatal responses. For example, UV-B counteracts far-red-induced ABA accumulation during seedling establishment, thereby modulating stomatal behavior under changing light environments (Gustavsson et al. 2026).

### Future perspectives on blue light signaling

Over the last decade, the core framework of blue light signaling in guard cells has been largely established: phototropins perceive light, BLUS1 functions as a guard-cell signaling kinase, CBC kinases suppress anion channel activity and phosphorylate the PM H^+^ ATPase, and the H^+^-ATPase drives membrane hyperpolarization and stomatal opening. However, critical gaps remain, particularly regarding the identity and regulation of the kinase(s) responsible for direct H^+^-ATPase phosphorylation of Thr948. Future studies combining phosphoproteomics, structural biology, and cell-type-specific genetic approaches will be essential to identify these missing components. Elucidating the complete kinase–phosphatase network will not only deepen our understanding of guard cell biology but also provide new strategies for manipulating stomatal behavior to improve water-use efficiency and stress tolerance in crops.

Blue light signaling does not operate in isolation but is integrated with other environmental and hormonal pathways. CO_2_, ABA, red light, humidity and heat signals all modulate guard cell behavior by acting on shared molecular targets such as ion channels and the PM H^+^-ATPase as reviewed in the following sections.

## Molecular mechanisms of abscisic acid triggered guard cell signal transduction and stomatal closing

Drought-induced abscisic acid biosynthesis has been found in the vasculature, roots, guard cells and possibly mesophyll tissues (Cornish and Zeevaart 1986; Christmann et al. 2005; Xie et al. 2006; Seki et al. 2007; Endo et al. 2008; Merilo et al. 2018). Early quantitative analyses provided evidence that both guard cells and other leaf cell types can cause ABA concentration increases in guard cells, but that water stress-induced quantitative contributions from guard cells were less than those from other leaf tissues (Cornish and Zeevaart 1986). Vasculature-derived ABA has been found to enable a rapid signal for drought-induced stomatal closure (Christmann et al. 2005; Seki et al. 2007; Endo et al. 2008; Merilo et al. 2018). In addition, roots produce ABA under soil drying conditions (Saab et al. 1990), and this root-derived ABA can be transported to the shoots to reinforce the drought response (Saab et al. 1990; Sauter et al. 2002). Notably, grafting studies in tomato and needle water potential measurements in *Callitris rhomboidea* demonstrated that root-derived ABA was not essential for stomatal closure, indicating possible species-dependent differences, but also relevantly tomato grafting implicated a root derived signal that differs from abscisic acid (Holbrook et al. 2002; Bourbia et al. 2026). The CLE25 peptide was identified as moving from roots to leaves and mediating stomatal closure in response to drought (Takahashi et al. 2018). Furthermore, a rapidly moving hydraulic signal from roots to shoots can report osmotic/drought conditions in roots and initiate ABA synthesis in leaves (Christmann et al. 2005).

An additional rapid production of ABA in *Arabidopsis* guard cells has been proposed via β-glucosidase1 (AtBG1)-mediated deconjugation of inactive ABA conjugates, including ABA-glucose ester (Lee et al. 2006). However, a subsequent study on double mutant plants in which both of the proposed *Arabidopsis* β-glucosidases, *AtBG1* and *AtBG2*, were knocked out, showed wildtype-like intact stomatal closing kinetics in response to ABA and to low relative air humidity in intact plants (Merilo et al. 2015). Further research, including intact plant stomatal conductance response analyses, will be needed to determine whether ABA deconjugation is relevant for rapid stress-induced stomatal closing and whether additional β-glucosidases might play a role or not.

Thus, both local leaf and vasculature synthesis contribute to the regulation of stomatal aperture during water stress, with root-derived ABA contributing, possibly dependent on the species (Wilkinson and Davies 2010). Abscisic acid initiates several protective mechanisms in plants against drought and desiccation (Finkelstein et al. 1998; Leung and Giraudat 1998; Cutler et al. 2010). The molecular mechanisms of the ABA-mediated signaling network have been extensively studied, and in guard cells rapid ABA-induced membrane potential changes, ion transport, and metabolic regulation act in an integrated fashion to bring about stomatal closure (Murata et al. 2015; Zhu and Assmann 2017).

Stomatal closure is triggered by various environmental and hormonal cues, which collectively regulate the activity of ion channels, ion pumps and metabolism in guard cells. In response to the stomatal closing signal abscisic acid, the plasma membrane H^+^-ATPase, which is highly active during stomatal opening, becomes dephosphorylated and inactivated (Zhang et al. 2004; Ando et al. 2022; Zait et al. 2025). Two types of anion channels in the plasma membrane of guard cells, the Slow and sustained (S-type) and Rapid (R-type), enable anion efflux and depolarization, which are required for stomatal closing (Keller et al. 1989; Schroeder and Hagiwara 1989; Schroeder and Keller 1992). Abscisic acid activates S-type anion channels in the plasma membrane of guard cells, triggering anion efflux (Pei et al. 1997). Anion efflux via S-type and R-type anion channel activation by ABA, together with proton pump inhibition, result in plasma membrane depolarization (Roelfsema et al. 2004). Anion efflux and the resulting plasma membrane depolarization serve as a central event in stomatal closure, as they also promote K^+^ loss via depolarization-activated K^+^ efflux (K^+^_out_) channels (Schroeder et al. 1987, 1993; Keller et al. 1989; Schroeder and Hagiwara 1989; Blatt 1990; Thiel et al. 1992). The combined release of anions and K^+^ increases the water potential of guard cells relative to the external space, thereby driving osmotic water efflux, and decreasing the turgor pressure of the guard cells resulting in stomatal closure.

### Binding of ABA to its receptor initiates OST1 kinase-dependent signal transduction

Early research showed that intracellular ABA is important for K^+^ channel regulation in guard cells (Schwartz et al. 1994). ABCG25 is an ATP-binding cassette (ABC) transporter that primarily functions as an ABA exporter. It is predominantly expressed in vascular tissues and localizes to the plasma membrane, where it mediates the transport of ABA from vascular tissues to target cells, including guard cells (Kuromori et al. 2010). ABCG40 is one of the mechanisms that can function as an ABA importer, facilitating ABA uptake into guard cells (Kang et al. 2010). ABA moves into guard cells and interacts with its receptors. ABA receptors are members of the PYRABACTIN RESISTANCE (PYR)/PYR-LIKE (PYL)/REGULATORY COMPONENTS OF ABA RECEPTORS (RCAR) family (Ma et al. 2009; Park et al. 2009). These PYR/RCAR receptors possess a highly conserved ABA-binding pocket, enabling perception of increases in ABA concentration under environmental stress (Melcher et al. 2009; Miyazono et al. 2009; Nishimura et al. 2009; Yin et al. 2009). PYR/PYL/RCAR receptors undergo a conformational rearrangement that allows them to interact with and inhibit type 2C protein phosphatases (PP2Cs), such as ABI1, ABI2, HAB1, and PP2CA, which act as negative regulators of ABA signaling (Merlot et al. 2001; Yoshida et al. 2006; Umezawa et al. 2009; Cutler et al. 2010) (Fig. 2). This inhibition releases SNF1-related protein kinases 2 (SnRK2s), such as OST1/SnRK2.6, SnRK2.2, and SnRK2.3, from PP2C-mediated repression. Activated SnRK2 kinases subsequently phosphorylate a wide array of downstream targets, including anion channels (SLAC1 and SLAH3) and the inward-rectifying K^+^ channel KAT1 (Geiger et al. 2009; Lee et al. 2009; Sato et al. 2009; Brandt et al. 2012; Acharya et al. 2013), transcription factors (AREB/ABFs), ROS-producing enzymes (RBOHF/D), other protein kinases and various metabolic enzymes leading to stomatal closure, gene expression reprogramming, and metabolic adjustment in response to drought and osmotic stress (Mustilli et al. 2002; Umezawa et al. 2009; Fujii et al. 2011; Brandt et al. 2015; Zhang et al. 2020; Shen et al. 2025) (Fig. 2). SnRK2 protein kinases phosphorylate and activate the S-type anion channels, SLAC1 and SLAH3, in *Xenopus* oocytes (Geiger et al. 2009; Lee et al. 2009; Brandt et al. 2012; Brandt et al. 2015; Qin et al. 2024). ABA activation of the SLAC1 anion channel has been reconstituted in *Xenopus* oocytes by co-expression of ABA receptors, the ABI protein phosphatase, the OST1 protein kinase and B3 family members of M3K/Raf-like protein kinases and SLAC1 (Brandt et al. 2012; Takahashi et al. 2020).

**Figure 2:**
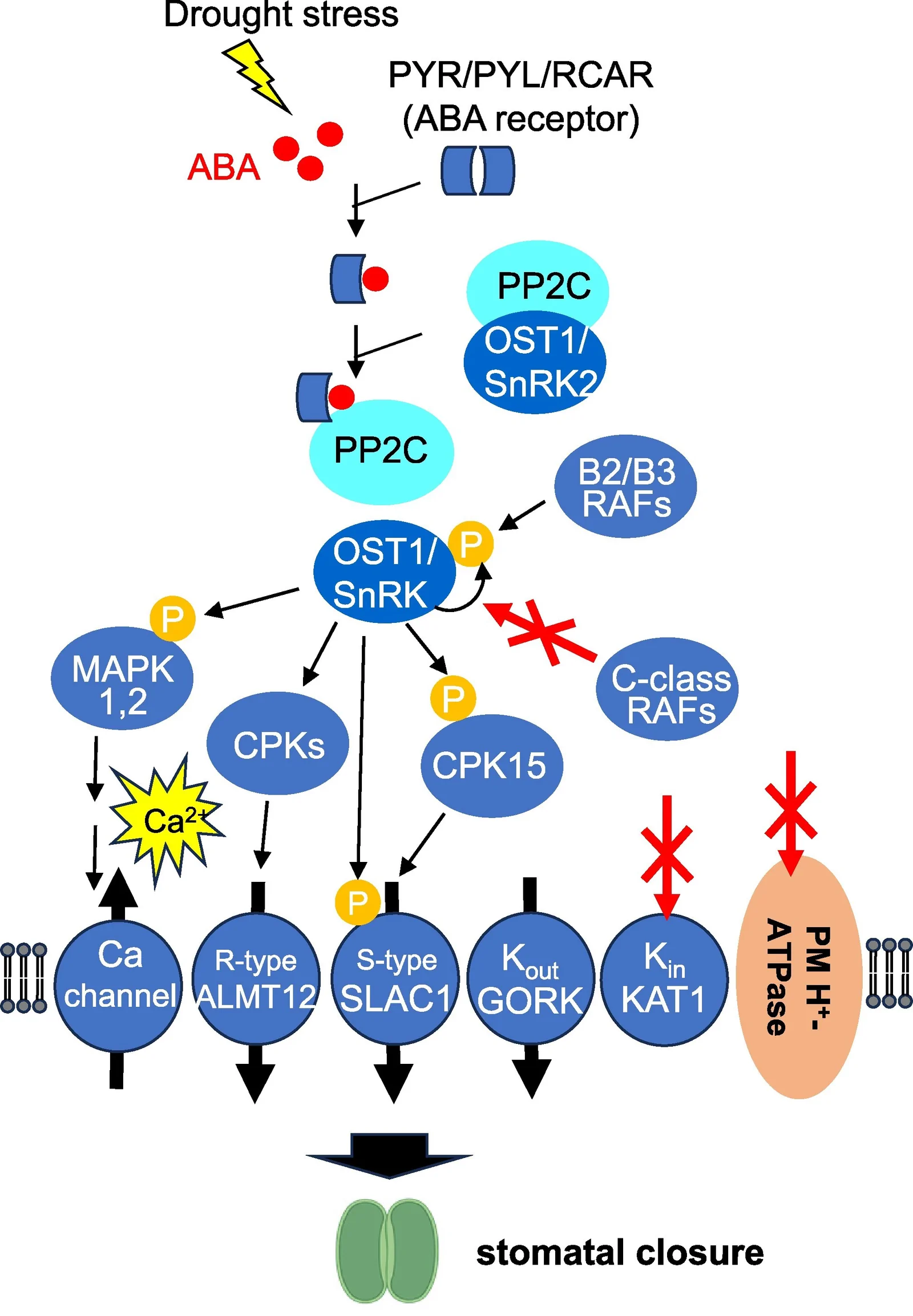
Early drought- and abscisic acid-induced stomatal closure signaling. Under drought conditions, abscisic acid (ABA) is synthesized and binds to ABA receptors in guard cells. This inhibits type A PP2Cs. PP2C inhibition enables phosphorylation of the OST1 protein kinase by B2/B3 Raf-like kinase (B2/B3 RAFs) thus activating the protein kinase OST1/SnRK2.6. OST1 phosphorylates and regulates many downstream proteins and initiates a phosphorylation cascade, including the indicated Ca^2+^-dependent protein kinases (CPKs) and MAPK kinases (MAPK1,2). Among ABA responses, this results in the activation of S-type and R-type anion channels and efflux of anions from guard cells. Anion channel-mediated depolarization activates K^+^_out_ channel-mediated K^+^ efflux from guard cells. ABA activation of Ca^2+^-permeable channels and inhibition of plasma membrane (PM) proton extruding ATPases contributes to this response. The loss of turgor causes guard cell shrinkage and stomatal closure.

### Hierarchical regulation of ABA signaling by RAF-like kinases and SnRK2s

In an additional layer of regulation, mitogen-activated protein kinase kinase kinases (M3Ks/RAF-like protein kinases) act as upstream activators and modulators of SnRK2s (Lin et al. 2020; Soma et al. 2020; Takahashi et al. 2020). It was previously considered that dephosphorylated inactive SnRK2/OST1 protein kinases could auto-activate themselves via auto-phosphorylation alone (Fujii et al. 2009; Ng et al. 2011). Unexpectedly however, dephosphorylation of OST1 in vitro, showed that OST1 could not reactivate itself solely through auto-phosphorylation (Takahashi et al. 2020). A subclade of MAPKKKs/Raf-like protein kinases was found to be the missing link. A forward genetic screen for ABA hyposensitivity using genomic scale microRNA-based silencing of closely homologous genes, identified members of the B3 clade of MAP kinase kinase kinases (M3Ks/Raf-like kinases) (Hauser et al. 2013). Further support for a role of this subclade of kinases in ABA signaling was obtained in *Physcomitrium patens*, where mapping of an ABA hyposensitive mutant identified a *Physcomitrium* homolog of the *Arabidopsis* B3 clade of M3K/Raf-like kinases (Saruhashi et al. 2015).

RAF-like kinases belonging to the B3 and B2 subgroups (such as M3Kɗ1(RAF3), M3Kɗ6 (RAF5/SIS8) and M3Kɗ7 (RAF4) are essential for the full activation of ABA-responsive SnRK2 kinases, including OST1/SnRK2.6 (Lin et al. 2020; Soma et al. 2020; Takahashi et al. 2020) (Fig. 2). These RAF-like kinases function as positive regulators that directly phosphorylate SnRK2s at conserved activation loop residues in response to osmotic stress and ABA accumulation (Lin et al. 2020; Soma et al. 2020; Takahashi et al. 2020). In contrast, C-class RAF-like kinases, including RAF36 and RAF22, act as negative regulators of ABA signaling by interacting with and suppressing SnRK2 activity, thereby preventing excessive ABA responses (Kamiyama et al. 2021). OST1/SnRK2.6 can phosphorylate RAF36 and RAF22, leading to their degradation via the 26S proteasome pathway (Kamiyama et al. 2021), suggesting the presence of a feedback loop that fine-tunes ABA sensitivity by balancing kinase activation and repression.

### Ion channels and membrane depolarization

Anion channel-mediated anion efflux is an early and critical step in stomatal closing, involving the release of Cl^−^ and organic anions such as malate. Classical studies demonstrated Cl^−^ and malate efflux from guard cells (Van Kirk and Raschke 1978a, 1978b; MacRobbie 1982). Subsequent research has also indicated that sugar metabolism and export contribute to osmotic adjustment in guard cells (Outlaw 2003; Santelia and Lawson 2016). In parallel, ABA induces cytosolic alkalization, which enhances the activity of outward-rectifying K^+^ channels (K^+^_out_ channels) and promotes K^+^ efflux to accelerate turgor loss (Irving et al. 1992; Blatt and Armstrong 1993; Miedema and Assmann 1996). pH-dependent activation occurs through membrane-localized mechanisms (Miedema and Assmann 1996). Recent structural and electrophysiological studies have advanced our understanding of K^+^ channel regulation by interacting factors, providing new insight into the molecular basis of ABA-dependent modulation (Jeanguenin et al. 2011; Clark et al. 2020; Li et al. 2025; Yamanashi et al. 2025; Zhang et al. 2025). Box 1 reviews some of the very early electrophysiological studies on plant cells, namely algal cells, including findings of similar properties of K^+^_out_ channels as well as some possibly unique channel properties found in guard cells (Box 1).

Box 1 A brief background on plant electrophysiology history: links to guard cells. While many of the advances in understanding early guard cell signal transduction have been started and/or driven by electrophysiological patch voltage clamp investigations, it is important to look at the historical development of plant electrophysiology in this context. Before the development of patch clamp techniques by Neher and Sakmann (Hamill et al. 1981) and before electrical recordings in guard cells, electrophysiological studies of plants identified electrical signals, in particular in large algal cells (Cole and Curtis 1938; Beilby and Coster 1979). Moreover, early studies including mechanical stimulation of *Mimosa pudica* (“sensitive plant”) and the Venus fly trap, led Francis and Charles Darwin to propose similarities between neurons and plant cells (Darwin and Darwin 1880). Later research showed the electrical mechanisms in neurons and plant cells to be largely distinct.

In the 1930s electrical recordings were pioneered on large algal cells, including *Nitella*, that showed action potentials, preceding many studies on neurons (Osterhout 1934; Cole and Curtis 1938). Further studies showed that these “action potentials” differed in their depolarization phase from the voltage-dependent sodium currents in animal action potentials, with evidence suggesting that permeabilities to chloride and calcium ions were predominant during the initial depolarization phase of algal action potentials to more positive membrane voltages (Gaffey and Mullins 1958; Hope and Findlay 1964).

The repolarization of action potentials to more negative membrane voltage is mediated by K^+^ efflux in both neurons and algal cells. Depolarization-activated outward-rectifying K^+^ (K^+^_out_) channels in animal cells systems and plant cells mediate this repolarization and have similar properties, albeit with slowed activation kinetics in algal cells and land plants (Findlay and Coleman 1983; Schroeder et al. 1987) compared with neurons. Voltage clamp recordings on giant algal cells were limited for technical reasons, but Findlay and Coleman adapted the freshwater alga *Hydridictyon africanum* for voltage clamp recordings revealing the presence of slowly activating outward-rectifying K^+^ channels (Findlay and Coleman 1983), similar to those later identified in *Vicia faba* guard cells (Schroeder et al. 1987). The inward-rectifying K^+^ “uptake” (K^+^_in_) channels were initially identified in guard cells (Schroeder et al. 1987), with many land plants cells utilizing this mechanism for low-affinity K^+^ uptake and as a “back-up” mechanism for high-affinity K^+^ uptake as well.

While investigating stomatal closing, MacRobbie identified chloride efflux from guard cells and Van Kirk and Raschke measured malate efflux from guard cells during stomatal closing (Van Kirk and Raschke 1978a, 1978b; MacRobbie 1982). These studies together led to the hypothesis and investigation that guard cells may use plasma membrane anion channels for both anion efflux and for depolarization (Schroeder and Hagiwara 1989), thus driving K^+^ efflux. Studies showed that guard cells have at least 2 major classes of plasma membrane anion channels (Schroeder and Keller 1992), the slowly and sustained activating “S-type” anion channels (Schroeder and Hagiwara 1989) and the rapidly and transiently activating “R-type” anion channels (Keller et al. 1989), that are required for ABA-induced stomatal closing (Schroeder et al. 1993; Schwartz et al. 1995). Thus, early electrophysiological studies on giant algal cells show certain similarities in general mechanisms identified in patch clamp investigations of guard cells and may be linked to similarities of algal chloride and Ca^2+^ currents to ion channels in land plants. While less is known about the genes and time-resolved activation properties of anion and Ca^2+^ channels in algal plasma membranes measured with voltage clamping, it would be interesting to investigate whether they are related to those in guard cells.

S-type anion channels are activated by phosphorylation events through ABA-activated protein kinases, including OST1 and Ca^2+^-dependent protein kinases (CPKs) (Schmidt et al. 1995; Li and Assmann 1996; Mori and Muto 1997; Li et al. 2000; Geiger et al. 2009; Lee et al. 2009; Geiger et al. 2010; Brandt et al. 2012; Brandt et al. 2015; Shen et al. 2025). The R-type anion channel ALMT12 (also known as QUAC1) contributes to transient, rapid anion currents and facilitates malate and Cl^−^ efflux, including during an initial phase of stomatal closure (Meyer et al. 2010; Sasaki et al. 2010). These anion channel activities are coordinated by Ca^2+^-dependent pathways mediated by CPKs and Ca^2+^-independent pathways primarily governed by the OST1 protein kinase. Supporting a role for Ca^2+^-dependent signaling, loss of function in Ca^2+^-activated protein kinases dampens induction of stomatal closure (Mori et al. 2006; Saito et al. 2018; Schulze et al. 2021).

### ABA-induced stomatal closure through Ca^2±^-independent and Ca^2±^-dependent phosphorylation

Early research suggested a role for calcium in ABA inhibition of stomatal opening (DeSilva et al. 1985). Classical studies revealed that ABA or CO_2_ concentration elevation cause increases in the cytosolic Ca^2+^ concentration (McAinsh et al. 1990; Webb et al. 1996). Early research showed that guard cells exhibit stimulus-induced oscillations in the cytosolic Ca^2+^ concentration, with data showing that increases in cytosolic Ca^2+^ correlated closely with the degree of stomatal closure (McAinsh et al. 1995). This and related research led to a Ca^2+^ signature hypothesis, in which the pattern of Ca^2+^ oscillations encodes the outcome of a cytosolic Ca^2+^ response. Evidence for this hypothesis in inhibition of stomatal re-opening came from research showing that, some imposed patterns of cytosolic Ca^2+^ elevations in guard cells, caused long-term “Ca^2+^-programmed” inhibition of stomatal re-opening (Allen et al. 2001; Cho et al. 2009). This programmed long-term Ca^2+^-induced stomatal closing was abolished by chelator-mediated removal of extracellular Ca^2+^, which also caused disruption of cytosolic Ca^2+^ transients, demonstrating the requirement of the imposed Ca^2+^ transients for this programmed inhibition of stomatal re-opening response (Figure S1 in (Allen et al. 2001). In contrast to this long-term Ca^2+^ programmed inhibition of stomatal re-opening (Figure S1 in Allen et al. 2001), rapid “Ca^2+^-reactive” stomatal closing was largely dependent on the total number of Ca^2+^ transients or total amount of experimentally imposed cytosolic Ca^2+^ elevations (Allen et al. 2001; Cho et al. 2009; Huang et al. 2024). These data indicate that the integral of cytosolic Ca^2+^ elevations within a time short period may control the percentage or rate of stomatal closing (Allen et al. 2001; Cho et al. 2009; Huang et al. 2024), which may to a degree be less dependent on the detailed pattern (signature) of cytosolic Ca^2+^ elevations.

Further research found that the stomatal closing stimuli ABA and high CO_2_ concentration (Young et al. 2006; Siegel et al. 2009) enhance or “prime” the intracellular cytosolic Ca^2+^ sensitivity of mechanisms that mediate stomatal closing (Siegel et al. 2009; Chen et al. 2010). In guard cells, physiological elevation in the cytosolic Ca^2+^ concentration caused only a weak regulation of anion and K^+^_in_ channels (Siegel et al. 2009; Chen et al. 2010). ABA and high CO_2_ enabled a strong cytosolic Ca^2+^ activation of S-type anion channel and cytosolic deactivation of K^+^_in_ channels in *Arabidopsis* and *Vicia faba* guard cells, allowing Ca^2+^ dependent stomatal closing primed by these biological stimuli (Siegel et al. 2009; Chen et al. 2010). Investigating the underlying molecular mechanisms in *Arabidopsis* guard cells showed that the above-described ABA receptor-mediated deactivation of clade A type-2 protein phosphatases (eg ABI1), is required for enabling cytosolic Ca^2+^-activation of S-type anion channels in guard cells (Brandt et al. 2015). Quadruple loss-of-function mutants in the PP2Cs ABI1, ABI2, HAB1 and PP2CA enabled strong cytosolic Ca^2+^ activation of S-type anion channels in guard cells without addition of ABA (Brandt et al. 2015). Thus, ABA primes the SLAC1 S-type anion channel activation in guard cells via PP2C inhibition, thereby enhancing cytosolic Ca^2+^ activation.

However, the above priming mechanism does not exclude additional Ca^2+^ sensitivity priming mechanisms and whether CPKs themselves are modified to enhance their Ca^2+^ sensitivity remained unknown. Recent research has shown that the OST1 protein kinase can activate SLAC1 via activation of the Ca^2+^-dependent CPK15 protein kinase (Shen et al. 2025). OST1 binds to and phosphorylates CPK15 at the CPK15-threonine-103 amino acid. Interestingly, this phosphorylation of CPK15 enhances the cytosolic Ca^2+^ sensitivity of CPK15 activation, providing an additional mechanism for ABA-induced Ca^2+^ sensitivity priming (Shen et al. 2025). CPK15 phosphorylates the SLAC1 N-terminus at 8 identified residues (Shen et al. 2025), which are known to activate SLAC1 (Geiger et al. 2009; Brandt et al. 2012; Qin et al. 2024). These studies together have led to an understanding of the molecular mechanisms mediating Ca^2+^ sensitivity priming of cytosolic Ca^2+^ signaling in guard cells (Brandt et al. 2015; Shen et al. 2025).

As cytosolic Ca^2+^ elevation in the same cell type can trigger different outputs and Ca^2+^ triggers numerous responses in plant cells, increasing (priming) the Ca^2+^ sensitivity of one of these cellular outputs can provide specificity of the Ca^2+^ response. Ca^2+^ sensitivity priming provides a mechanism for specificity in Ca^2+^ signaling, ensuring that other Ca^2+^-induced signaling pathways do not cause unintended responses. Ca^2+^ sensitivity priming mechanisms (Brandt et al. 2015; Shen et al. 2025), could also not require as strict of a biological control of the precise pattern (signature) of the Ca^2+^ elevations in guard cells for Ca^2+^ reactive stomatal closure, which may be more dependent on the integrated cytosolic Ca^2+^ elevation within a short time period as discussed above.

Additional mechanisms could support crosstalk between Ca^2+^-dependent and Ca^2+^-independent signaling pathways. OST1 phosphorylates MAP4K1 and MAP4K2, which were reported to activate downstream Ca^2+^ channels and promote stomatal closure (Yamashita et al. 2025). These data indicate an additional link through which OST1 may function as a molecular link between the Ca^2+^-independent and Ca^2+^-dependent pathways in ABA-regulated stomatal closure.

In contrast to CPKs, in a distinct class of Ca^2+^ responsive protein kinases is the “CBL-CIPK” class. When all 5 plasma membrane-targeted Ca^2+^-binding Calcineurin B-like proteins (CBLs) were mutated in the *cbl1/4/5/8/9* quintuple mutant, stomatal closing and stomatal opening responses in intact plants were indistinguishable from those of wild type plants (Schulze et al. 2021). CBL-CIPKs have been found by heterologous expression in *Xenopus* oocytes to activate SLAC1 via phosphorylation (Maierhofer et al. 2014). However, subsequent research showed that CIPK23 promotes stomatal opening rather than stomatal closing (Huang et al. 2023). These findings provide an example that caution needs to be taken in interpreting kinase regulation of plant transporters in oocytes for predicting in vivo functions in guard cells (Maierhofer et al. 2014). It remains to be analyzed in intact plant gas exchange analyses which physiological stomatal closing stimuli, and whether these oocyte expression findings, may translate to a role *in planta* in plasma membrane SLAC1-mediated stomatal closing. For vacuolar membranes, other CBLs with CIPKs, CBL2/CBL3—CIPK9/CIPK17 complexes, localize to the vacuolar membrane, where they regulate vacuolar ion transport and thus subcellular redistribution of K^+^ and anions, contributing to the fine-tuning of cellular osmotic balance during ABA-induced responses (Weinl and Kudla 2009; Batistic et al. 2010).

The above reviewed research and previous research is consistent with a model that Ca^2+^ and several CPKs have an accelerating function in controlling the speed of stomatal closing and opening in intact *Arabidopsis* plants (Huang et al. 2019; Schulze et al. 2021). The dynamic crosstalk between the Ca^2+^-independent SnRK2 pathway and Ca^2+^ dependent signaling has been proposed to enable plants to fine-tune stomatal responses to ABA and environmental stimuli (Kudla et al. 2018). Collectively, calcium-mediated phosphorylation pathways constitute a multilayered regulatory network that modulates both plasma membrane and vacuolar transport activities, ensuring precise temporal and spatial control of guard cell turgor and stomatal movements.

### ABA activation of Ca^2+^ channels in guard cells

ABA is known to activate Ca^2+^-permeable cation channels in the plasma membrane of guard cells, thereby enhancing Ca^2+^ influx (Hamilton et al. 2000; Pei et al. 2000). The resulting Ca^2+^ signal plays a key role in accelerating downstream ABA responses, as described above. As these Ca^2+^-permeable channels are largely activated at more negative (hyperpolarized) membrane potentials, they may be mainly relevant at membrane potentials before guard cells are depolarized (Hamilton et al. 2000; Pei et al. 2000). Loss-of-function mutants in certain calcium-dependent protein kinases, like *cpk3* and *cpk6*, show impaired activation of these ABA-induced Ca^2+^-influx channels in guard cells, indicating that these CPKs may act in a positive feedback loop to amplify the Ca^2+^-based signaling cascade (Mori et al. 2006). In this view, when ABA initially activates Ca^2+^ influx, the elevated cytosolic Ca^2+^ may further activate CPKs, which in turn would enhance Ca^2+^ influx channel activity and downstream targets, thus reinforcing the signal. Additionally, reactive oxygen species (ROS) have been found as intermediates linking ABA perception to Ca^2+^ channel activation: ABA induces H_2_O_2_ production in guard cells, which can directly gate Ca^2+^-permeable channels (Pei et al. 2000; Foreman et al. 2003). This ROS–Ca^2+^ coupling may provide an extra amplification step upstream of Ca^2+^ signaling. Furthermore, ROS activation of Ca^2+^-permeable channels in guard cells and other cell types may provide a mechanism by which oxidative stress rapidly activates Ca^2+^ signaling. Additional mechanisms that function in guard cell ABA and/or in CO_2_ signal transduction, including reactive oxygen species production and signaling by other second messengers, including NO, have been extensively reviewed elsewhere (Murata et al. 2015; Kudla et al. 2018).

Much of the detailed research into molecular and cellular mechanisms of abscisic acid signal transduction in stomatal guard cells has been conducted with *Arabidopsis* and *Vicia faba*, with some interesting findings made in other species. Elucidating species-specific characteristics and potential differences in guard cell signal transduction could be directly linked to understanding plant strategies for environmental adaptation. Further research, including analyses of natural variants and analyses leveraging mapping populations, can advance this research towards other species.

### Novel ABA receptor candidates

Other candidate ABA receptors have been reported; one recent candidate is the rice nitrate transporter NRT1.1B, which has been proposed to function as an ABA receptor (Ma et al. 2025). NRT1.1B in rice is 1 of the 2 most closely related genes to the *Arabidopsis* nitrate transporter NRT1.1/CHL1. Time-resolved ABA-regulated stomatal conductance analyses in intact plants (Lopez et al. 2024) will be needed to assess the relative contribution of NRT1.1B to ABA-induced stomatal closing. Further research is needed to determine whether and how a potential diversity of ABA receptors might provide information to explain ABA signaling.

### CO_2_ sensing and signal transduction in guard cells

The atmospheric carbon dioxide (CO_2_) concentration is predicted to double during the present century. Elevated CO_2_ reduces stomatal apertures of plants globally, with increasingly profound effects on global plant transpiration, water use efficiency, leaf heat stress and plant robustness, including in crops and trees (Medlyn et al. 2001; Franks et al. 2017). CO_2_ regulation of stomatal movements plays a major role in continental and terrestrial water cycles (Hungate et al. 1997; Betts et al. 2007; Berry et al. 2010; Jasechko et al. 2013). The CO_2_ concentration inside leaves falls and rises daily due to photosynthesis and respiration in the dark, triggering stomatal movements, with an added impact of the atmospheric CO_2_ rise (Medlyn et al. 2001; Franks et al. 2017). A molecular, genetic and biochemical understanding of stomatal CO_2_ sensing and signal transduction has been advanced in recent years.

### CO_2_ regulation of guard cell ion channels

Elevated CO_2_ activates S-type and R-type anion channels in guard cells (Roelfsema et al. 2002; Raschke et al. 2003; Xue et al. 2011; Hsu et al. 2018) and enhances the activity of the outward-rectifying K^+^ efflux channels (Brearley et al. 1997). Furthermore, elevated CO_2_ dephosphorylates the plasma membrane H^+^-ATPase including at the penultimate C-terminal residue, resulting in inhibition of proton pump activity (Ando et al. 2022). Together this network of plasma membrane ion channels and pump regulation mediates high CO_2_-induced plasma membrane ion efflux from guard cells, turgor reduction and stomatal closing.

### Starch metabolism

In addition to rapid CO_2_ regulation of transport processes, CO_2_ regulates guard cell starch synthesis and degradation (Flütsch et al. 2020). Elevated CO_2_-induced stomatal closure is attenuated in guard cell starch biosynthesis mutants but not in mesophyll starch biosynthesis mutants (Azoulay-Shemer et al. 2016). These findings are consistent with a requirement to remove osmotically active malate via synthesis of osmotically inactive starch in guard cells (Schnabl et al. 1982; Outlaw and Tarczynski 1984). In line with this notion, starch degradation in guard cells (Flütsch et al. 2020) contributes to low CO_2_-induced stomatal opening in *Arabidopsis* (Silva-Alvim et al. 2026).

### Ca^2+^ regulation of the speed of stomatal CO_2_ responses

Early studies suggested a role for calcium in high CO_2_-induced stomatal closing (Schwartz et al. 1988; Webb et al. 1996). Cytosolic Ca^2+^ increases were found to rapidly activate S-type anion channels and downregulate K^+^ uptake (K^+^_in_) channels in *Vicia faba* guard cells, which provides mechanisms mediating inhibition of stomatal opening and promotion of stomatal closing (Schroeder and Hagiwara 1989). Further analyses unexpectedly indicated a role for cytosolic Ca^2+^ elevations in both CO_2_-regulated stomatal closing and stomatal opening in intact plants (Young et al. 2006). Analyses of higher order Arabidopsis mutants in Ca^2+^-dependent protein kinases (CPKs) found that *cpk3/5/6/11/23* quintuple mutants slowed elevated CO_2_-induced stomatal closure, and remarkably also markedly slowed low CO_2_-induced stomatal opening (Schulze et al. 2021). These phenotypes were not observed in other investigated single *cpk* mutant alleles, 2 related *cpk* quadruple mutants nor a different *cpk* quintuple mutant.

The slowing but not disrupting of the stomatal CO_2_ responses in *cpk3/5/6/11/23* quintuple mutant plants may indicate that either further CPK/Ca^2+^-binding protein mutants are needed or that the role of calcium lies in regulating the speed of stomatal closing (Schulze et al. 2021). This latter hypothesis is supported by studies in which cytosolic calcium plays a modulatory role in regulating the degree or kinetics of stomatal closing (Siegel et al. 2009; Huang et al. 2019; Schulze et al. 2021).

### β-Carbonic anhydrases

Despite elucidation of the above-discussed downstream signaling elements, the question remained of how plants sense and respond directly to CO_2_. Reverse genetic analysis of guard cell and mesophyll-expressed known CO_2_- and bicarbonate-binding proteins identified double mutant plants in the β-carbonic anhydrases 1 and 4 (βCA1 and βCA4) as slowing the kinetics of high CO_2_-induced stomatal closing (Hu et al. 2010; Hu et al. 2015). A recent study has shown that βCA4 binds directly to the SLAC1 anion channel, thereby activating SLAC1 and suggested that the carbonic anhydrase activity is not required for SLAC1 activation (Xia et al. 2026). Carbonic anhydrases are nature's fastest known catalytic enzymes and have evolved at least 4 distinct forms. Guard cell expression of a structurally distinct mammalian cytosolic α-carbonic anhydrase II (αCAII) was found to complement the CO_2_ response of *βca1 βca4* double mutant in intact plants, providing evidence that carbonic anhydrase-mediated catalytic activity is an important mechanism for βCA-mediated CO_2_-induced stomatal closure (Hu et al. 2010; Hu et al. 2015). In addition, catalytically active chloroplast-targeted carbonic anhydrases in rice and cytosolic-localized carbonic anhydrases in maize and Arabidopsis are sufficient to mediate CO_2_ control of stomatal movements, indicating that catalytically inactive CAs at the plasma membrane might not always be required (Hu et al. 2015; Chen et al. 2017; Kolbe et al. 2018). Further research will be needed to determine the relative contribution of inactive carbonic anhydrase to CO_2_-induced stomatal closing. Another study isolated a mutant in the catalytically active α-carbonic anhydrase-7 that impaired CO_2_-induced stomatal closing in epidermal peels (Sun et al. 2022).

Patch clamp analyses, structure-function analysis of the S-type anion channel SLAC1 and biochemical analyses further provided evidence that bicarbonate is an important intracellular second messenger in guard cells that mediates the CO_2_ response (Hu et al. 2010; Xue et al. 2011; Takahashi et al. 2022). Together these data suggested that an important role of the β-carbonic anhydrases in guard cells lies in accelerating the catalysis of CO_2_ to bicarbonate and protons (Hu et al. 2010; Hu et al. 2015). β-carbonic anhydrase mutants in rice and maize showed similar slowing of stomatal CO_2_ responses, suggesting that β-carbonic anhydrases are of general relevance for the rate of CO_2_-triggered stomatal movements (Chen et al. 2017; Kolbe et al. 2018) (Fig. 3).

**Figure 3:**
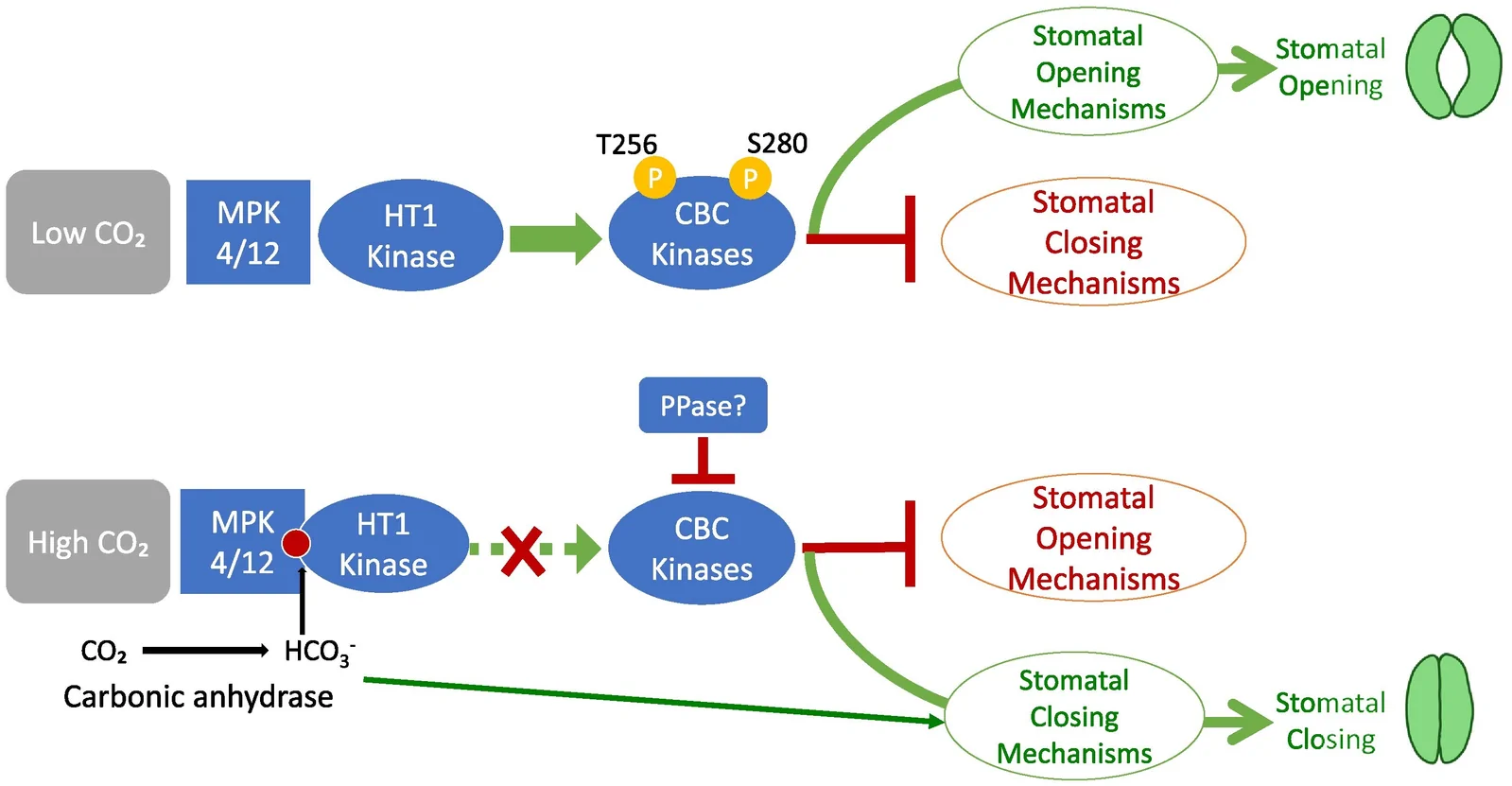
Guard cell CO_2_ sensing core regulation of stomatal apertures. Simplified model showing: Top graphic: At low CO_2_, the HT1 Raf-like protein kinase is active and phosphorylates and activates the CBC kinases. CBC kinase activation leads to downstream phosphorylation of target proteins that mediate stomatal opening and that inhibit stomatal closing. Bottom graphic: CO_2_ elevation increases the bicarbonate (HCO_3_^−^) concentration in guard cells facilitated by β-carbonic anhydrases. Elevated CO_2_/bicarbonate enhances the interaction of the HT1 protein kinase with the MAP kinase 4 and MAP kinase 12 (MPK4/12). MPK4/12 and HT1 together form the primary sensor of CO_2_/bicarbonate in guard cells, with these proteins being essential for the stomatal CO_2_ response. The high CO_2_/bicarbonate-induced MPK–HT1 interaction inhibits the HT1 kinase activity. HT1 kinase inhibition, together with unknown protein phosphatases that would dephosphorylate CBC kinases, cause downregulation of CBC protein kinase activities, thus mediating high CO_2_-induced stomatal closing mechanisms and downregulating stomatal opening mechanisms. In addition, β-carbonic anhydrases cause enhancement in the activity of the S-type anion channel SLAC1 as secondary CO_2_/bicarbonate sensing mechanism, thereby facilitating high CO_2_/bicarbonate-mediated stomatal closing.

Double knock out of *βCA1* and *βCA4* slowed, but did not disrupt the stomatal closing and opening response to elevated CO_2_ (Hu et al. 2010; Hu et al. 2015). The findings that stomatal CO_2_ responses are slowed, but not disrupted in carbonic anhydrase mutants in intact maize, rice and Arabidopsis plants indicated that (ⅰ) carbonic anhydrases are not primary CO_2_ sensors (contrary to a proposed model [Xia et al. 2026]), and (ⅱ) that other “primary” CO_2_ sensors that regulate stomatal movements are likely to exist. Structure-function analysis of a direct bicarbonate-increased activity of SLAC1 anion channels provided *in planta* evidence that SLAC1 provides a “secondary” CO_2_/bicarbonate sensor, with a primary CO_2_ sensor likely regulating upstream protein kinases (Chater et al. 2015; Zhang et al. 2018) (Fig. 3). Atomic scale resolution of SLAC1 structures provided further evidence for this model in which the C- and N-terminal domains deactivate the SLAC1 channel by folding onto and plugging the transmembrane domain of SLAC1, which includes interactions with the bicarbonate-neutralized residue R256 (Li et al. 2022a, 2022b).

It has been stated that the added bicarbonate concentrations that increase SLAC1 activity (Zhang et al. 2018) represent “100 times atmospheric CO_2_” and are therefore nonphysiological (Blatt et al. 2022; Xia et al. 2026). However, this estimate is misleading, as the concentrations of solubilized CO_2_ and bicarbonate quickly equilibrate with the surrounding ambient CO_2_ concentration in lab air (partial CO_2_ pressure in air) and depend on the solution pH and temperature, as described by Henry's law (Sander 2015). Therefore, it is incorrect to conclude (Xia et al. 2026) that experimentally added bicarbonate salts represent the final CO_2_/HCO—concentrations in solution (see (Sander 2015) for more information). Rather, adding bicarbonate salts to an open solution will see a rapid decline in the free bicarbonate concentration that will eventually equilibrate with the surrounding air, rather than being set to the stated incorrect “100 times atmospheric CO_2_” (Xia et al. 2026).

Nevertheless, it has been discussed that higher CO_2_/bicarbonate concentrations may be required in vitro, if “an additional mechanism such as a HCO_3_^−^ concentrating mechanism, unknown scaffold, other factors, local compartmentation mechanisms, and/or posttranslational modifications of [the CO_2_ sensor described below] may be active *in planta*” (Takahashi et al. 2022). The finding that SLAC1 activity can be enhanced by bicarbonate elevation (Zhang et al. 2018), that mutations that inhibit this bicarbonate regulation impair biological stomatal response in intact leaves at physiological CO_2_ concentrations (Zhang et al. 2018) and that physiological CO_2_ concentration elevation of 800 ppm activates S-type anion channels in guard cells (Hsu et al. 2018), further pointed to the fact that the primary protein kinase-regulating CO_2_/HCO_3_^−^ sensors in guard cells remained unknown.

### Primary stomatal CO_2_ sensing via MAP-kinase—HT1 Raf-kinase protein-protein regulation

Forward genetic screening of EMS-mutagenized Arabidopsis plants identified the RAF-like protein kinase High Temperature1 (HT1) (Hashimoto et al. 2006). *ht1* mutant alleles that abrogate kinase activity show completely disrupted low CO_2_-induced stomatal opening, and show a constitutive high CO_2_ response (low stomatal conductance). The MAP kinase *MPK12* was identified, including through natural variation mapping in Arabidopsis for water use efficiency (Jammes et al. 2009; Des Marais et al. 2014; Jakobson et al. 2016). MPK12 was found to primarily control the CO_2_ response (Jakobson et al. 2016; Tõldsepp et al. 2018). Double *mpk12 mpk4GC* mutant alleles were generated, in which *MPK4* is silenced specifically in guard cells. These *mpk12 mpk4GC* double mutant alleles strongly impair high CO_2_-induced stomatal closing, but not ABA-induced stomatal closing (Tõldsepp et al. 2018).

Thus, the stomatal CO_2_ response phenotypes of *ht1* and *mpk12 mpk4GC* double mutant plants were the strongest CO_2_ insensitive mutants known, but with opposing phenotypes (*ht1*: low stomatal conductance and complete low CO_2_ insensitivity; *mpk12 mpk4GC* double mutant: high stomatal conductance and insensitivity to elevated CO_2_ [Hashimoto et al. 2006; Tõldsepp et al. 2018]). The recombinant proteins MPK12, MPK4 and HT1 alone in solution did not show kinase activity up- or downregulation as a function of the added CO_2_/bicarbonate in vitro (Tõldsepp et al. 2018; Takahashi et al. 2022), implying that the primary CO_2_ sensor that regulates these protein kinases may lie further upstream. Unexpectedly however, combined genetic, biochemical and physiological studies found that the protein complex of HT1 together with MPK12/4 functions as a primary CO_2_/bicarbonate sensor in guard cells (Takahashi et al. 2022) (Fig. 3). Elevated CO_2_/bicarbonate enhances the interaction of HT1 with MPK12/4 resulting in direct inhibition of the HT1 protein kinase activity, with corresponding stomatal responses at physiological CO_2_ concentrations *in planta* (Takahashi et al. 2022). Note that, unexpectedly, protein kinase activity of MPK12 was found not to be required for this CO_2_-mediated MPK12-HT1 interaction, HT1 kinase inhibition and for the *in planta* stomatal CO_2_ response (Takahashi et al. 2022).

### Stomatal CO_2_ signaling in *Brachypodium* grass

Grasses have a distinct stomatal morphology, consisting of dumbbell-shaped guard cells and neighboring subsidiary cells (Raissig et al. 2016). Unbiased forward genetic infrared thermal imaging screening of an EMS-mutagenized population of the grass *Brachypodium distachyon* recently mapped a high CO_2_ insensitive mutant, *chill1*, to the single *Brachypodium* ortholog of MPK12 and MPK4, BdMPK5/Chill1 (Lopez et al. 2024). Stomatal conductance of the *chill1* mutant remains larger than wildtype controls upon transition to CO_2_ elevation, resulting in cooler leaf temperatures. CRISPR disruption *BdMPK5/Chill1* alleles showed a complete insensitivity to high CO_2_-induced stomatal closing but not ABA-induced stomatal closing. Similarly, silencing of a related MAP kinase in *Nicotiana tabacum* caused CO_2_-insensitive but functional ABA-regulated stomatal movements (Marten et al. 2008). Together these studies provide independent confirmation of the primary role of this MAP kinase in CO_2_ signaling and together with in vitro biochemical CO_2_ response assays and structural modeling of BdMPK5 with BdHT1 indicate a similar CO_2_ sensing mechanism in this grass species and *Arabidopsis* (Lopez et al. 2024) (Fig. 3).

### Convergence of low CO_2_ signaling with blue light signaling

As discussed earlier for guard cell blue light signaling, the Convergence in Blue light and CO_2_ (CBC) signaling Raf-like protein kinases, CBC1 and CBC2, were identified and shown to be genetically required for both blue light- and low CO_2_-induced stomatal opening (Hiyama et al. 2017). Double mutant alleles in *CBC1* and *CBC2* show a low stomatal conductance (ie a constitutive high CO_2_ response) and strong impairment in low CO_2_-induced stomatal opening (Hiyama et al. 2017). CBC1 and CBC2 are in a different clade than HT1 among the ∼80 Raf-like kinases encoded in the *Arabidopsis* genome. The *Arabidopsis* HT1 kinase was found to phosphorylate and activate the CBC1 kinase at low CO_2_ (Takahashi et al. 2022). Mutation of 2 HT1-phosphorylated sites of CBC1 to alanine disrupted complementation of the stomatal low CO_2_ response of the *cbc1 cbc2* double mutant at physiological [CO_2_] *in planta*, providing biochemical and genetic evidence that the MPK12/4—HT1 CO_2_ sensor transmits low CO_2_-induced stomatal opening by activation of the CBC kinase (Takahashi et al. 2022).

### Distinct early high CO_2_ sensing and ABA receptor cores in mediating stomatal closing

A model has been proposed that elevated CO_2_ signals via early ABA receptor signal transduction (Dittrich et al. 2019). However, follow-up research could not confirm some of the key findings (Zhang et al. 2020). Furthermore, activation of S-type anion channels at 800 ppm CO_2_ in guard cells and stomatal closing were not disrupted in higher order quadruple, quintuple and hextuple ABA receptor mutants (Hsu et al. 2018). Moreover, while CO_2_ elevation closed stomata within ∼20 min, no increases in the guard cell ABA concentration were observed within 1 h of high CO_2_ exposure in intact guard cells using both real-time ABA-FRET and transcriptional reporters (Hsu et al. 2018).

Further evidence for a separation of early ABA and CO_2_ signaling in guard cells was obtained by investigating chromatin remodeling in guard cells in response to these stimuli (Seller and Schroeder 2023). ABA was found to dramatically and differentially regulate chromatin accessibility in guard cells, mesophyll cells and root cells, including at numerous 5′UTRs of genes that are up and downregulated by abscisic acid (Seller and Schroeder 2023). In contrast, CO_2_ only very moderately regulated chromatin accessibility in guard cells with statistically different predicted 5′ responsive elements compared with the ABA response in guard cells (Seller and Schroeder 2023).

### So how do CO_2_ and ABA interact and why are their early signaling mechanisms separated?

Despite the above findings, both elevated CO_2_ and ABA stimulate stomatal closing and regulate the same endpoints in guard cells (eg ion channels, pumps and metabolism). Indeed, classical studies found that elevated CO_2_ increases the ABA-induced rate and degree of stomatal closing in time-resolved stomatal conductance analyses of intact *Xanthium strumarium* leaves, with less strong CO_2_ effects on the stomatal ABA response in *Gossypium hirsutum* and *Commelina communis* (Raschke, 1975a, 1975b). Similarly, high CO_2_-induced stomatal closing is greatly amplified by ABA synthesis and ABA signaling (Hsu et al. 2018; Movahedi et al. 2021). Interestingly, findings indicate that even under nonstress conditions, the ABA concentration in guard cells is higher than in other leaf cells, and basal ABA signaling amplifies the stomatal CO_2_ response (Lahr and Raschke 1988; Hsu et al. 2018; Zhang et al. 2020). Both in gel kinase assays of OST1 activity in guard cells and a real time OST1/SnRK2 protein kinase activity FRET reporter, “SNACS”, found that in contrast to ABA exposure, high CO_2_ did not rapidly activate OST1/SnRK2 protein kinases (Hsu et al. 2018; Zhang et al. 2020). However, these real-time kinase activity FRET imaging analyses also provided evidence that OST1/SnRK2 protein kinases have a basal activity in intact guard cells (Zhang et al. 2020), consistent with the basal levels of ABA found in nonstressed guard cells (Lahr and Raschke 1988; Hsu et al. 2018). Together these findings point to the model that basal ABA and basal ABA signal transduction greatly amplify early CO_2_ signaling.

But why are early CO_2_ sensor and ABA reception pathways in first order distinct—notwithstanding likely transcriptional crosstalk? Recent findings point to a model in which ABA reprograms the guard cell genome and signaling in response to drought, as a form of guard cell stress memory, based on findings that stomata will usually not re-open for a few days after rewatering drought-stressed plants (Seller and Schroeder 2023). In contrast, stomatal CO_2_ signaling is known to trigger rapid reversible stomatal opening and closing. This occurs for example upon cloud cover and sun re-emergence when C_i_ rapidly changes, in addition to direct blue light and red light signaling pathways. Thus, while CO_2_ (C_i_) shifts in leaves cause rapid reversible stomatal responses, high ABA concentrations program longer term stomatal closing. Further research is needed to determine how and where the high CO_2_ and ABA signal transduction pathways converge, enabling regulation of the same downstream ion channels, pumps and guard cell metabolism.

### Open questions in CO_2_ sensing and signaling

Several open questions remain in addition to those discussed above, including whether, where and how CO_2_ and/or bicarbonate bind and increase the affinity of the HT1 interaction with MPK12/MPK4 (Takahashi et al. 2022). At high CO_2_ the present model predicts that the CBC kinases should be inhibited via dephosphorylation (Fig. 3). Protein phosphatases that deactivate CBC1 and/or CBC2 in vivo remain to be identified. MPK12/MPK4, HT1 and the CBC kinases form an early CO_2_ signaling core (Fig. 3). However, it remains unclear how the kinase activity of CBC kinases regulates the plethora of downstream ion channels, pumps and other intracellular signals that regulate stomatal opening. An integrated Boolean model provides insights and testable hypotheses for investigating how the CO_2_ sensing core can interact with and regulate diverse signaling mechanisms, second messengers and transporters in guard cells (Gan et al. 2024). One model suggests that CBC kinases may directly downregulate the S-type anion channel SLAC1 (Hiyama et al. 2017). Recent findings reveal a direct regulation of the H^+^-ATPase by CBC1 in complex with HT1 (Takase et al. 2026).

## Red light stimulation of stomatal opening, models and mechanisms

### The enigmatic stomatal response to red light

Because the magnitude of red light-induced stomatal opening exceeds that of the phototropin-mediated blue light response, the red light response is of paramount importance in the regulation of carbon gain and water use efficiency. The study of this guard cell response has had a long and checkered history. A major challenge in identifying the intrinsic guard cell response to red light is that the same wavelengths of light that open stomata also drive photosynthesis. This congruency has raised challenges in determining whether there is a direct guard cell response to red light independent of the guard cell response to the reduction in intercellular CO_2_ that arises from mesophyll photosynthesis. A 1954 porometry study by Heath and Russell (1954) on the responses of wheat stomata to (white) light in which CO_2_ free air was forced through the leaf to eliminate carbon assimilation concluded, “… it may be said that the experiment described in the present paper tends to confirm the suggestion of a light effect *independent of carbon dioxide* seen in Heath and Milthorpe's (1950) data (Heath 1950), but leaves undecided the question of whether it is entirely *an indirect effect transmitted from epidermal or mesophyll cells by some unspecified agent travelling from cell to cell*, or whether it is in part due to a *direct light response in the guard cells* themselves of the kind postulated by those authors.” In short, this early study left open possibilities (see italicized phrases; emphasis added) that guard cells were responding to a mesophyll signal *other than* CO_2_, and that guard cells were directly responding to light. Remarkably, as explicated in Box 2 and below, these same themes appear in subsequent research up until the present day.

Box 2 Early studies on the guard cell response to red light. The early history of research into stomatal responses to light was compiled by Hans Meidner in 1987 (Meidner et al. 1987). Here, only selected early studies of particular relevance to the red light response are briefly summarized. One of the key early studies on stomatal responses to light in epidermal peels—in which any influence of the mesophyll is necessarily absent—was performed by Pieter Kuiper in 1964 (Kuiper 1964). He obtained an action spectrum for the maintenance of stomatal opening in *Senecio odoris*. His data revealed that blue light was more effective than red light, while green light was largely ineffective. However, based on his observations that DCMU could cause stomatal closure under white light, he hypothesized that guard cell chlorophyll was the (sole) photoreceptor responsible for stomatal responses to light.

In 1966, Terry Mansfield and Hans Meidner investigated stomatal opening in *Xanthium pennsylvanicum* (cocklebur; now *Xanthium strumarium*) and were among the first to remark on the greater efficiency of blue light in stimulating stomatal opening at low light intensities (Mansfield and Meidner 1966). In 2 key papers published in 1981 (Sharkey and Raschke 1981, 1981), Thomas Sharkey and Klaus Raschke obtained detailed action spectra of stomatal conductance in *Xanthium strumarium* and other species. They confirmed the greater effectiveness of blue light in promoting stomatal conductance, and the similarity of the red light action spectrum to that of photosynthesis. They also observed a severe reduction of stomatal conductance under red light upon application of the photosynthetic inhibitors, DCMU and cyanizine, but only a moderate decrease in stomatal conductance under blue light. Through leaf inversion experiments, in which they observed that stomatal conductance was greater when light directly impinged on the epidermis, vs. being filtered by the mesophyll as in the inverted situation, they concluded that the photoreceptors for both the red light response and the blue light response were likely to be located in the guard cells. These researchers may have been the first to explicitly state (Sharkey and Raschke 1981) “*Probably 2 photoreceptors are involved”*, although this conclusion was presaged by Hsiao, Allaway, and Evans in 1973 (Hsiao et al. 1973), whose action spectra on malate formation in guard cells in *Vicia faba* epidermal peels in the presence of saturating background red light illumination led them to propose the presence of a blue light photoreceptor other than chlorophyll.

Additional studies on isolated epidermal peels of *Vicia faba* and *Commelina communis* by Amnon Schwartz and Eduardo Zeiger (Schwartz and Zeiger 1984) confirmed the greater efficacy of blue light over red light in stimulating stomatal opening. Similar to Hsiao et al. (1973) they used dual beam protocols, which demonstrated that, under background red light intensities at which stomatal opening plateaued, further stomatal opening could be evoked by addition of blue light. In both plant species, these researchers also observed that DCMU resulted in elimination of red light-induced stomatal opening but only partial inhibition of blue light-induced stomatal opening, supporting the idea of a photoreceptor for blue light distinct from chlorophyll. Note that while here we use the convenient shorthand of the “red light” response to distinguish it from the blue light specific (ie phototropin-mediated) response, chlorophyll also absorbs blue light; thus, DCMU also partially inhibited the blue light response in their study and others (Suetsugu et al. 2014), as expected for a chlorophyll-mediated response. Around the same time, research in the Zeiger lab (Zeiger et al. 1983) revealed that stomata in epidermal peels from *Paphiopedilum harrisianum*, an orchid that lacks guard cell chlorophyll, do not open in response to red light, but do so in response to blue light. These experiments reinforced the ideas that the guard cell red light (PAR) photoceptor is chlorophyll, and that the specific blue light response is mediated by a photoreceptor other than chlorophyll. These early observations, despite the lack of molecular and genetic tools, provided valuable information for modern investigations of the red light response.

While the studies summarized in Box 2 would seem to firmly establish a direct guard cell response to red light that is mediated by guard cell chlorophyll, these concepts have not gone unchallenged by more contemporary studies. For example, 2 studies from the Hedrich lab in the early 2000s concluded that the appearance of a red light response of guard cells actually arose from the reduction in intercellular CO_2_ concentrations (c_i_) as a result of mesophyll photosynthesis, based on their observations that: (ⅰ) focused red light illumination of guard cell pairs failed to induce ion transport characteristics associated stomatal opening, while red light illumination of a larger area of the leaf, and reductions in c_i_, did (Roelfsema et al. 2001, 2002), and; (ⅱ) a failure of red light to open stomata in albino patches of leaves (Roelfsema et al. 2006), leading to their conclusion that the “red light” response of guard cells actually is a response to a decrease in c_i_. However, around the same time, Keith Mott and colleagues (Messinger et al. 2006) refuted this conclusion. Analogous to the 1952 study of Heath and Russell, they performed experiments in which *c_i_* was held constant and found that stomata in intact leaves of *Xanthium strumarium* still opened in response to red light.

Some of these apparent contradictions are at least partially resolved by a study from the Assmann lab (Zhu et al. 2020), which showed that the magnitude of the red light response, at least in *Arabidopsis*, is impacted by growth conditions: plants grown under low light intensities (0.125 mmol m^−2^s^−1^) and low humidity exhibit weak to absent red-light-induced stomatal opening in epidermal peels, as compared to plants grown under high light intensities (0.300 mmol m^−2^s^−1^) and higher humidity. By contrast, *Vicia faba* plants (also grown at 0.125 mmol m^−2^s^−1^ and low humidity) exhibit clear red light-induced stomatal opening. These results demonstrate that choice of species and growth conditions can strongly impact the ability to observe the direct guard cell response to red light, a topic worthy of further study.

It is also accurate that stomatal apertures in epidermal peels under red light are smaller than those in intact leaves, and that red light activation of the guard cell H^+^-ATPase is small (Serrano et al. 1988) or below the detection limit (Assmann et al. 1985; Taylor and Assmann 2001) in isolated protoplasts, suggesting that there is a role of the mesophyll in red light-induced stomatal opening. Considering the entirety of the literature, the results indicate that the “red light response” of stomata in the intact leaf actually consists of 3 components: (ⅰ) an indirect response to red light, mediated by light-induced changes in c_i_; (ⅱ) a direct, chlorophyll-mediated and environmentally-tuned (Zhu et al. 2020) response of guard cells to red light; (ⅲ) a non-CO_2_-dependent role of the mesophyll in enhancing the guard cell red light response (Mott 2009) (as nicely summarized by Mott in 2009). Below, current understanding of mechanisms #2 and #3 are discussed. Guard cell responses to CO_2_ have been reviewed above. The paradigmatic mechanism of stomatal opening has been established largely from studies on the blue light-specific response, wherein it has been shown that light-dependent phosphorylation of plasma membrane H^+^-ATPases (encoded in Arabidopsis by *AHA* genes) increases their activity (Fig. 1) (Shimazaki et al. 1986; Hayashi et al. 2010, 2011). Consequent H^+^ extrusion hyperpolarizes the membrane (Assmann et al. 1985), thereby activating voltage-gated K^+^ channels that mediate K^+^ influx (Schroeder et al. 1987). Uptake of other solutes also occurs, for example via symport with H^+^, along with closure of anion channels, that mediate anion efflux. These phenomena result in a net increase in guard cell solutes that drives water influx, cell swelling, and stomatal opening (Fig. 1).

Recent studies suggest that a largely analogous regulation mechanism of the AHA H^+^-ATPase underlies red light-induced stomatal opening. Toshinori Kinoshita and colleagues showed that a null mutant of AHA1, *aha1-9*, revealed retarded stomatal opening over the first 60 min of red light exposure in whole leaves (Ando and Kinoshita 2018). Stomatal apertures eventually reached wild-type levels (Ando and Kinoshita 2018), suggesting contributions from other AHA family members (Ando and Kinoshita 2018). The team next applied immunofluorescence techniques to quantify phosphorylated AHA in guard cells, and showed that red light (and blue light) stimulated phosphorylation of the AHA-Thr948 residue (also known as pen-Thr), the penultimate AHA-threonine residue in guard cells of intact leaves, while only blue light-induced phosphorylation was observed in guard cells isolated in epidermal peels.

It was subsequently shown that Thr881 in guard cells in epidermal peels and guard cell protoplasts, in the absence of the mesophyll, is a target of phosphorylation under both blue light and red light (Fuji et al. 2024; Hayashi et al. 2024). The phosphorylation of Thr881 in isolated epidermal peels and guard cell protoplasts was inhibited by DCMU (Fuji et al. 2024; Hayashi et al. 2024), clearly implicating guard cell chloroplasts in this guard-cell-autonomous red light response (Fig. 1). *aha1-9* lines complemented with a nonphosphorylatable version of AHA1, AHA1-T881A, exhibited impairment in both stomatal opening in epidermal peels and stomatal conductance increases following blue + red light exposure, confirming that this phospho-site was regulatory of stomatal opening. To summarize, red light exposure of intact leaves leads to phosphorylation of Thr948 of the AHA H^+^-ATPase, while red light reception by guard cell chloroplasts triggers phosphorylation of Thr881 in AHA1. Phosphorylation promotes H^+^ ATPase activity, which drives stomatal opening.

### Phytochrome involvement in the red light response?

Given a role for red light in stomatal responses, it is natural to ask whether the red/far-red photoreceptor, phytochrome, is involved in red light-induced stomatal opening. A role for both guard cell-localized and mesophyll-localized phytochrome in control of guard cell development and stomatal density has been demonstrated (Casson et al. 2009; Kang et al. 2009; Casson and Hetherington 2014). But data on whether phytochrome plays a role in rapid stomatal aperture responses are equivocal.

An early study by Roth-Bejerano and Itai (1981) reported that simultaneous far-red illumination reduced the extent of red light-induced stomatal opening in epidermal peels of *Commelina communis*, and also promoted Rb^+^ (as a proxy for K^+^) loss from guard cells, seemingly in strong support for a role of phytochrome. In Arabidopsis, a slight reduction in stomatal apertures of *phyB* null mutants relative to wild-type was observed, albeit only at 1 of the 4 fluence rates of red light tested; the far-red reversibility of this phenomenon was not queried (Wang et al. 2010). Wang and colleagues (Wang et al. 2017) also proposed negative feedback regulation of PHYB-dependent stomatal opening that is mediated by the guanine nucleotide exchange factor, RopGEF2, and the small GTPases, ROP2 and ROP7, and a kinase cascade involving MKK2 and MPK2 also has been implicated by genetic analyses (Li et al. 2023).

In contrast to these results, Zait et al. (2025) saw no difference between wild-type and *phyB* knockouts in red light-induced stomatal opening in isolated Arabidopsis epidermal peels. Sharkey and colleagues observed no effect on stomatal conductance in Arabidopsis of simultaneous far-red illumination in the presence of red light, regardless of whether the genotype was wild-type, a *phyB* null, or plants with *PHYB* knocked down specifically in guard cells or mesophyll cells (Weraduwage et al. 2022). Moreover, Ando and Kinoshita observed that Arabidopsis *phyA phyB* double mutants were identical to wild-type in their levels of red light-induced Thr948 (pen-Thr) phosphorylation (Ando and Kinoshita 2018).

Just as for red light-induced stomatal opening (Zhu et al. 2020), the plant growth environment may play a role in whether far-red reversal of stomatal opening is observed. Assuming that light conditions during growth modulate chloroplast development in guard cells in a partially phytochrome-dependent manner, as they do in mesophyll cells (Dubreuil et al. 2017), prior growth conditions might render stomatal opening more or less susceptible to far-red impacts on red light-induced stomatal opening, without any direct role of phytochrome in the rapid stomatal red light response. This would also be consistent with the reduced basal levels of stomatal conductance reported for *phyB* deficient plants (Weraduwage et al. 2022). Finally, well-documented impacts of *PHYB* knockout on whole-plant development (Tripathi et al. 2019) may also indirectly influence the stomatal response to red light by altering signaling metabolites emanating from the mesophyll, which are discussed below.

### Guard cell ATP

Assays of guard cell ATP levels in earlier studies showed that these levels are reduced by DCMU, or in guard cells that lack chloroplasts, indicating that a role of the guard cell chloroplasts is to provide energy for the H^+^ ATPase, as well as, potentially, provide the reactant (ie ATP) necessary for kinase-mediated AHA phosphorylation to occur (Tominaga et al. 2001). For example, in 2001, Ken-ichiro Shimazaki and colleagues showed in guard cell protoplasts of *Vicia faba* that DCMU inhibited red light-promotion of guard cell ATP levels, while in 2014 Wang et al. (2014) studied the *crumpled leaf* (*crl*) mutant of Arabidopsis, which has a mosaic of guard cells containing and lacking chloroplasts, and found that the latter had lower ATP levels and less stomatal opening under (white) light than the former. Energy may also be provided by guard cell photosynthesis in forms other than ATP: guard cells in isolated *Vicia faba* epidermal peels opened and accumulated sucrose when exposed to red light (Talbott and Zeiger 1993), implicating photosynthetic production of sugars that could serve as both an osmoticum and an energy source. However, it is not clear that chloroplast-produced ATP or sugars are rate limiting for stomatal opening: antisense inhibition of cytb6f (Baroli et al. 2008), rubisco small subunit (Baroli et al. 2008), or SBPase (Lawson et al. 2008) in tobacco did not impair stomatal conductance responses to red light.

### Mesophyll messengers

The fact that Ando and Kinoshita (2018) observed that under red light, phosphorylation of the AHA1-Thr948 residue (pen-Thr) occurred in intact leaves but not in isolated epidermis hinted at a role for the mesophyll in this process (Fig. 1). As early as 1979, Suan Wong, Ian Cowan, and Graham Farquhar had noted a close relationship between rates of mesophyll photosynthesis and stomatal conductance (Wong et al. 1979), introducing the concept of a “mesophyll messenger”. This hypothesis was strengthened by experiments in the 1990s in which media supplemented with supernatant from mesophyll cells exposed to red but not blue light promoted swelling of guard cell protoplasts of *Commelina communis* (Lee and Bowling 1993). In the 21st century, modern metabolomics methods provided the requisite tools to document and dissect red light influences on the apoplastic metabolome (Zait et al. 2025). Accordingly, Yotam Zait, Toshinori Kinoshita, Sixue Chen, Sarah Assmann and colleagues used HPLC to fractionate apoplast fluid and identify fractions that enhanced red light-induced stomatal opening in both *Arabidopsis thaliana* and *Vicia faba* epidermal peels. They then applied metabolomics approaches to identify small molecules present in the active fractions, identifying over 400 metabolites in total. Metabolites that were elevated by red light in apoplastic fluid from both species were tested for their efficacy in enhancing red light-induced stomatal opening in epidermal peels and in enhancing red light-induced stomatal conductance when fed through the petiole. Of these metabolites, sugars, particularly sucrose, at the same (µM – low mM) concentrations as those naturally prevailing in the red light apoplast, emerged as mesophyll messengers (Fig. 1). At high concentrations, externally applied sucrose had an inhibitory effect on stomatal opening, because of the resultant osmotic gradient opposing guard cell water influx. Of interest, Bill Outlaw and colleagues in the early 2000s (Kang et al. 2007) had proposed accumulation of sucrose in the guard cell apoplast under high transpiration conditions as a mechanism that would promote stomatal closure and thus limit plant water loss under dehydrating atmospheric conditions.

One role of apoplastic sucrose, if subsequently imported into guard cells, could be as an energy source (Flütsch and Santelia 2021). The presence of the mesophyll was previously reported to increase guard cell ATP levels, although the underlying mechanism was not elucidated (Wang et al. 2014). However, Zait et al. (2025) found that apoplastic sucrose failed to induce stomatal opening in the dark, indicating that sucrose is not simply relieving an energy limitation on stomatal opening, and consistent with a signaling role of extracellular sucrose (Zait et al. 2025). This led to investigation of possible modulation by extracellular sucrose of guard cell ion transport.

Biochemical and electrophysiological studies employing extracellular sucrose revealed that this mesophyll messenger at concentrations naturally found in red light-illuminated leaves, enhances stomatal opening via phospho-activation of the H^+^ ATPase and inhibition of anion channel opening in guard cells (Zait et al. 2025). The underlying signaling cascades await elucidation. Potential regulatory mechanisms of other apoplastic metabolites, including maleic acid (Zait et al. 2025), and other organic acids (Hedrich and Marten 1993; Araujo et al. 2011; Zait et al. 2025) are intriguing topics for future study.

### Open questions in red light sensing and signaling

Further elucidation of the intracellular signaling cascade that links red light perception in the guard cell chloroplast with plasma membrane ion transport, identification of additional extracellular factors that modulate the red light response, and any exact role of phytochrome in the guard cell red light response are compelling topics for future research.

## Vapor pressure difference regulation of stomatal movements

Low atmospheric humidity and/or an increase in temperature cause an increase in the vapor pressure difference between the inner spaces of leaves to the air surrounding leaves (VPD_leaf_). Low relative air humidity, corresponding to an increase in VPD_leaf_, triggers rapid stomatal closing within ∼10 to 20 min, thereby reducing transpirational water loss (Mott and Parkhurst 1991; Buckley 2005; Xie et al. 2006). Leaf water potential is an important determinant of stomatal behavior (Bourbia et al. 2026). The molecular mechanisms mediating high VPD_leaf_-induced stomatal closing have received renewed interest in recent years with many open questions. Advances and hypotheses have been reviewed (Mills et al. 2024) and we briefly review recent mechanistic advances and models here.

High VPD_leaf_-induced stomatal closing in angiosperms is mediated by a combination of 2 general pathways: a slow passive hydraulic stomatal response (hydro-passive) and a signal transduction-requiring “hydro-active” response (Merilo et al. 2018; Buckley 2019). In the hydro-passive response, low humidity for example causes water loss from guard cells resulting in low turgor of guard cells and stomatal closing. For the hydro-active response, signal transduction is implicated, since increased VPD_leaf_-induced stomatal closing is impaired in *ost1* protein kinase mutants and guard cell anion channel double mutants (*slac1/almt12*) and in other mutants of guard cell signaling components (Xie et al. 2006; McAdam et al. 2016; Merilo et al. 2018; Hsu et al. 2021; Zait et al. 2024).

Rapid ABA production in guard cells has been proposed as an early primary signal in the low humidity/high VPD_leaf_ stomatal closing response (Xie et al. 2006; Bauer et al. 2013; McAdam and Brodribb 2016; Sussmilch et al. 2017). Guard cell promoter-driven ABA biosynthesis in the ABA deficient mutant *aba3-1* was shown to rescue stomatal closing (Bauer et al. 2013). On the other hand, expression of enhanced ABA synthesis in phloem companion cells showed increased leaf temperatures and reduced water loss from detached leaves (Kuromori et al. 2014). As cell type- targeted promoters, can allow a level of expression in other leaf cells in some transgenic lines, it will be important to ascertain models, including from other guard cell complementation studies with fluorescently-tagged proteins to test for transgenic protein expression in other cell types. Using fluorescent protein fusions, complementation of ABA biosynthesis mutants showed that high VPD_leaf_ (low humidity)-induced stomatal closing could be restored by both phloem companion cell expression or guard cell expression (Merilo et al. 2018). From present studies, abscisic acid synthesis can occur in multiple tissues and further research could examine their quantitative contributions to the high VPD_leaf_ response.

A related question of present interest is, when does ABA synthesis become relevant during high VPD_leaf_ (low humidity)-induced stomatal closing? Using excised leaves exposed to high pressure using a Scholander pressure chamber, increases in the ABA concentration in leaves were detected after 20 min with further increased ABA levels found after 60 min (McAdam and Brodribb 2016). Exposing excised leaves to an external pressure of 1.5 MPa resulted in increased mRNA levels of the ABA biosynthesis gene 9-cis-epoxycarotenoid dioxygenase 3 (*NCED3*) (Sussmilch et al. 2017).

While ABA is known to play a role in several stomatal closing responses, further research showed that ABA-independent mechanisms play a role in the initial rapid stomatal closing response to an increase in VPD_leaf_ in intact plants (Assmann et al. 2000; Merilo et al. 2018; Yaaran et al. 2019). High VPD_leaf_-induced stomatal closing was functional in *Arabidopsis* and tomato ABA biosynthesis mutants, conditionally in *ost1* mutant plants and in ABA receptor hextuple mutants (Merilo et al. 2018; Zait et al. 2024). Moreover, severe ABA biosynthesis *Arabidopsis* mutants showed a similar rapid or slightly delayed rate of stomatal conductance drop to a VPD_leaf_ increase as wildtype controls (Merilo et al. 2018; Hsu et al. 2021).

How can this difference in findings be explained (Assmann et al. 2000; McAdam and Brodribb 2016; Sussmilch et al. 2017; Merilo et al. 2018)? Stomata close rapidly in response to a VPD_leaf_ increase in intact plants within 10 to 20 min (Merilo et al. 2018; Hsu et al. 2021). The time course of *NCED3* transcript upregulation, ABA concentration increase and stomatal conductance changes should be investigated in leaves of intact plants under identical rapid VPD shifts to determine the temporal sequence of events. Does exposure of excised leaves to external high pressure (McAdam and Brodribb 2016; Sussmilch et al. 2017) sufficiently mimic the in vivo VPD_leaf_ stimulus? The question remains whether ABA rises rapidly in response to a VPD_leaf_ increase in intact plants or later than the initial rapid hydroactive stomatal closing response? Alternatively, might high basal ABA concentrations in nonstressed guard cells (Lahr and Raschke 1988; Hsu et al. 2018) contribute to stomatal aperture regulation?

The question remains, if ABA concentration changes do not fully account for the rapid high VPD_leaf_ response (Assmann et al. 2000; Merilo et al. 2018; Yaaran et al. 2019), what are other early hydro-active signal transduction mechanisms? Mutants in the OST1/SnRK2.6 protein kinase conditionally impair the hydro-active stomatal closing response (Merilo et al. 2018; Hsu et al. 2021; Zait et al. 2024), further implicating additional pathways in the VPD_leaf_ response. A model has been proposed in which OST1 may be activated via an ABA-independent pathway—in addition to a possible (basal) ABA-dependent amplification (Merilo et al. 2018). A recent study found that the G protein mutants, *gpa1*, *agb1* and a G protein subunit quadruple mutant cause a robust impairment in high VPD_leaf_-induced stomatal closing (Zait et al. 2024), providing a genetic opening into a VPD signaling pathway.

The B3 and B4 sub-families of MAPKKK (M3 K/Raf-like) protein kinases have been shown to activate SnRK2 protein kinases, including OST1 via an osmotic stress pathway parallel to ABA (Lin et al. 2020; Soma et al. 2020; Takahashi et al. 2020). Recent research showed that a M3 K/Raf-like kinase quadruple mutant in the B3 sub-family of these protein kinases slows the high VPD_leaf_ response (Hsu et al. 2021). Whether members of another B4 sub-family, that contribute to the osmotic stress response, further enhance the impairment of the VPD_leaf_ response, remains to be determined. Furthermore, whether a VPDl_eaf_ increase activates these Raf-like/M3K kinase activities in guard cells, as was found for osmotic stress in whole seedlings (Lin et al. 2020; Soma et al. 2020; Takahashi et al. 2020), remains to be determined.

An additional interesting response of stomata to low humidity (high VPD_leaf_) is an initial transient stomatal opening response, followed by the above discussed rapid stomatal closing. This response was first reported about 100 yr ago, and is termed the “wrong way response” or “Ivanoff effect” (Ivanoff 1928). Research has provided evidence that low humidity first leads to hydro-passive water and turgor loss in the epidermal pavement cells neighboring guard cells, and this reduces the “backpressure” on guard cells, resulting in the transient opening of stomata (Mott and Franks 2001; Buckley 2005). A first mutant that clearly impairs this wrong way VPD_leaf_ response, as well as the subsequent stomatal closing, was recently identified in mutant alleles of GHR1 (Hsu et al. 2021). GHR1 encodes a guard cell-expressed plasma-membrane LRR receptor-like protein that functions in reactive oxygen species- and ozone-mediated stomatal closing (Hua et al. 2012; Sierla et al. 2018). GHR1 is an interesting transmembrane protein, for which mutants impair many known stomatal closing signal transduction pathways (Hua et al. 2012; Sierla et al. 2018; Hsu et al. 2021). Furthermore, the closest homolog to GHR1 in maize, PAN2, is expressed in stomatal subsidiary cells but not in guard cells and plays essential roles in communication of guard cells with their neighboring subsidiary cells in maize stomatal development (Zhang et al. 2012; Liu et al. 2024). These findings in maize together with impairment in the wrong way response in *Arabidopsis* has led to hypothesis that GHR1 may play a role in guard cell-to-neighboring pavement cell communication (Zhang et al. 2012; Hsu et al. 2021; Liu et al. 2024). In summary, interesting open questions remain as outlined above for understanding the mechanisms of low humidity/high VPD_leaf_-induced stomatal closing. The recent findings of G protein, Raf-like kinase and GHR1 receptor-like proteins (Hsu et al. 2021; Zait et al. 2024) having important functions in mediating high VPD_leaf_-induced stomatal closing, provide a foothold to dissect this relevant pathway in times of increasing global temperatures, humidity extremes and associated VPD increases.

## High temperature-mediated stomatal opening

High temperatures are important environmental factors that strongly influence stomatal movements and plant water relations. Physiological studies by Darwin (1898) and Klaus Raschke and colleagues demonstrated that heat can induce stomatal opening, suggesting that stomata actively respond to temperature signals in addition to light, CO_2_, and humidity (Raschke 1975a, 1975b). Stomatal opening can provide a mechanism for transpirational cooling of aerial plant organs. Subsequent studies modeled (Mott and Peak 2013) and provided molecular genetic evidence that warm temperatures (eg 28 °C) promote stomatal opening through enhanced photosynthetic activity, leading to reduced intercellular CO_2_ concentration (Ci) and thereby activation of the MPK4/MPK12 -HT1 CO_2_ sensor pathway (Pankasem et al. 2024).

However, when temperatures rise further, heat reduces photosynthetic activity, while stomata continue to open, demonstrating an uncoupling of stomatal responses from photosynthesis (Urban et al. 2017; Pankasem et al. 2024). Thus, warm temperatures and heat stress use 2 distinct signal transduction pathways that mediate stomatal opening (Pankasem et al. 2024), with new studies having started to uncover molecular genetic mechanisms required for the stomatal heat stress response.

Recent studies suggest that blue-light signaling components contribute to heat-induced stomatal opening. In particular, phototropins (phot1 and phot2), originally identified as blue-light phototropins receptors, have been linked to heat-induced stomatal opening (Kostaki et al. 2020). A working model proposes that heat signaling converges on the PM H^+^-ATPase activation pathway, which functions as a central hub integrating multiple environmental inputs. Recent work has expanded this model by identifying a direct molecular link between heat signaling and PM H^+^-ATPase activation. MAP4K4/TOT3 was reported to directly phosphorylate the penultimate threonine residue (Thr948) of the AHA1 H^+^-ATPase during heat-induced stomatal opening, thereby activating the PM H^+^-ATPase (Xu et al. 2025).

In addition to signaling pathways upstream of the PM H^+^-ATPase, temperature-dependent regulation of other ion transporters and metabolic regulation can contribute to stomatal responses. For example, temperature-sensitive regulation of K^+^ channels shows modulation of ion channel activities that can affect guard cell osmotic adjustment during heat responses (Ilan et al. 1995). Recent findings provide initial insights into how heat signals are transduced to stomatal opening, with further investigations being important given the increase global temperatures and occurrence of heat waves.

## Integrating signaling pathways

As the wealth of information on guard cell signaling grows, modeling approaches provide 1 path forward for comprehensive integration and interpretation of experimental observations. The OnGuard platform from Michael Blatt, Adrian Hills, and associates (Nguyen et al. 2023) takes inputs of environmental and leaf conditions and predicts information on stomatal conductance, assimilation, and transpiration along with numerous cellular parameters including stomatal aperture, guard cell solute content and transporter activity. OnGuard is a mechanistic model, and information on how the model's nonlinear differential equations were parameterized using experimental data from dicot systems is described in previous publications cited in Nguyen et al. (2023). OnGuard modeling has been used, for example, to guide mutation of the guard cell outward K^+^ channel, GORK, a manipulation that was found to slightly accelerate stomatal closure kinetics in response to a light-dark transition (Horaruang et al. 2022).

A different approach is provided by discrete dynamic modeling. In this approach, a network of components is first constructed and then Boolean operators (AND, OR, NOT) are applied to define how each node in the network is influenced by the upstream components to which it is connected. Discrete dynamic modeling is particularly useful when insufficient quantitative information is available for parameterization; it also allows facile integration of information on dozens of individual components that are often evaluated in isolation; both of these considerations apply to many of the intracellular signaling events studied in guard cells. Discrete dynamic modeling has been applied by the Albert and Assmann groups to model both ABA- and CO_2_-induced stomatal closure, and to predict interacting effects of ABA, red light, blue light, and CO_2_ on stomatal opening (Li et al. 2006; Sun et al. 2014). This modeling approach has, for example, predicted calcium inhibition of PP2C phosphatase activity in ABA-induced stomatal closure (Maheshwari et al. 2019), as well as a role of nitric oxide in CO_2_-induced stomatal closure (Gan et al. 2024); both of these predictions were then experimentally verified.

## Conclusions

Stomatal opening and closing are critical for plant survival and of relevance for understanding environmental ecology as well as for agricultural productivity in times of increasing stresses and weather extremes. Stomatal movements balance plant carbon gain with water loss. Stomatal aperture changes are regulated by a remarkably diverse array of environmental and biological signals, including blue and red light, carbon dioxide, humidity, water status, phytohormones, heat and pathogens. Although these stimuli often originate from distinct sensors and early signaling pathways, they ultimately converge on a common set of guard cell signaling components that control ion transport, guard cell metabolism, turgor changes, and thus finally stomatal apertures. Guard cells were among early plant biological systems in which single cell studies provided advanced insights into mechanisms and physiology, and remain a powerful model system for dissecting early sensing and signal transduction mechanisms in plants.

Stomatal response regulation is orchestrated by a highly integrated cellular network. Research advances over the past many years and recent breakthroughs have revealed key mechanisms within the stomatal signal transduction network, as we have reviewed here. It is clear that extensive crosstalk among signaling pathways must exist. Insights into crosstalk mechanisms have come from studying critical nodes in the guard cell signaling network, such as receptors for blue light and abscisic acid and the OST1/SnRK2 protein kinases. However, despite significant progress at identifying key molecular signal transducers and negative regulators and some convergent mechanisms, important questions remain regarding how guard cells integrate these multiple simultaneous signals under environmental stress or fluctuating natural conditions.

In this review, we have included some of the key historical background of identifying physiological aspects of stomatal regulation, while focusing on advances at understanding the intricate molecular mechanisms through which key biological stimuli and increasingly relevant stressors control the closing and opening of stomatal pores in land plants. Important open questions remain for each of the discussed biological and environmental stimuli, as highlighted in this review. We hope this review will help inspire future investigations. The emergence of genomics-inspired investigations of natural variation in diverse plant species will likely be of key relevance and lead to future conceptual and applied advances. Future studies combining molecular genetics, physiological, genomic, biophysical, and systems biology approaches will be essential for understanding the dynamic coordination of stomatal opening and closing and for harnessing this knowledge to improve plant productivity and resilience to weather extremes.
